# Supplementary material for: Detection of High Level of Co-Infection and the Emergence of Novel SARS CoV-2 Delta-Omicron and Omicron-Omicron Recombinants in the Epidemiological Surveillance of Andalusia
Source: Int J Mol Sci. 2023 Jan 26;24(3):2419. doi: 10.3390/ijms24032419 (PMC9916856; doi:10.3390/ijms24032419)
Supplement: Supplementary file 1 [file ijms-24-02419-s001.zip › ijms-2118634-supplementary.pdf]

Detection of high level of co-infection and the emergence of novel SARS-CoV-2 delta-omicron and omicron-omicron recombinants in the epidemiological surveillance of Andalusia

Supplementary Material

Content

Table S1. Sequences used in this study 1

Table S2. SARS-CoV-2 sequences compatible with co-infections 5

Table S3. Potential SARS-CoV-2 recombinant.. 27

Table S4. Sequences of the family in which the emergence of a recombinant was observed... 40

Table S5. Detailed Lolliplots of the sequences of the family in which the emergence of a recombinant was observed.. 41

The Andalusian COVID-19 Sequencing Initiative... 44

Table S1. Sequences used in this study

Sample	Event (C=Coinfection, R=Recombinant)	Collection date	Localization (Hospital and Province)	ENA Sample ID
AND17788	C	2022-01-12	Hospital Universitario San Cecilio (Granada)	SAMEA110395582
AND17799	C	2022-01-12	Hospital Universitario San Cecilio (Granada)	SAMEA110395583
AND19065	C	2022-01-17	Hospital Universitario Virgen de la Victoria (Málaga)	SAMEA110395584
AND19148	C	2022-01-22	Hospital Universitario Virgen de la Victoria (Málaga)	SAMEA110395585
AND19238	C	2022-01-28	Hospital Universitario San Cecilio (Granada)	SAMEA110395586
AND19468	C	2022-01-30	Hospital Universitario San Cecilio (Granada)	SAMEA110395587
AND19571	C	2022-02-02	Hospital Costa del Sol (Marbella, Málaga)	SAMEA110395588
AND20157	C	2022-02-04	Hospital Universitario Puerta del Mar (Cádiz)	SAMEA110395589
AND20304	C	2022-02-18	Hospital Universitario Puerta del Mar (Cádiz)	SAMEA110395590
AND20601	C	2022-02-15	Hospital Universitario de Jaén (Jaén)	SAMEA110395591
AND20905	C	2022-03-01	Hospital Universitario San Cecilio (Granada)	SAMEA110395592

AND20906	C	2022-03-01	Hospital Universitario San Cecilio (Granada)	SAMEA110395593
AND20966	C	2022-02-18	Hospital Virgen de la Victoria (Málaga)	SAMEA110395594
AND21001	C	2022-02-15	Hospital Universitario Virgen de la Victoria (Málaga)	SAMEA110395595
AND21137	C	2022-02-25	Hospital de Antequera (Málaga)	SAMEA110395596
AND21237*	R	2022-03-02	Hospital Universitario Reina Sofía (Córdoba)	SAMEA110395597
AND21266	R	2022-03-09	Hospital Universitario Virgen del Rocío (Sevilla)	SAMEA110395598
AND21375	C	2022-03-01	Hospital Universitario Puerta del Mar (Cádiz)	SAMEA110395599
AND21391	C	2022-03-08	Hospital Universitario San Cecilio (Granada)	SAMEA110395600
AND21405	C	2022-03-09	Hospital Virgen de las Nieves (Granada)	SAMEA110395601
AND21422	C	2022-03-04	Plataforma Provincial de Almería (Almería)	SAMEA110395602
AND21474	C	2022-03-01	Hospital Universitario de Jaén (Jaén)	SAMEA110395603
AND21475	C	2022-02-28	Hospital Universitario Virgen de la Victoria (Málaga)	SAMEA110395604
AND21483	C	2022-02-22	Hospital Universitario Virgen de la Victoria (Málaga)	SAMEA110395605
AND21520	C	2022-02-07	Hospital Universitario de Jaén (Jaén)	SAMEA110395606
AND21532	R	2022-03-07	Hospital Universitario San Cecilio (Granada)	SAMEA110395607
AND21550	C	2022-03-07	Hospital Universitario San Cecilio (Granada)	SAMEA110395608
AND21552	C	2022-03-07	Hospital Universitario San Cecilio (Granada)	SAMEA110395609
AND21554	C	2022-03-07	Hospital Universitario San Cecilio (Granada)	SAMEA110395610
AND21702	C	2022-02-04	Hospital Universitario Puerta del Mar (Cádiz)	SAMEA110395611
AND21845	C	2022-03-14	Plataforma Provincial de Almería (Almería)	SAMEA110395612
AND21903	C	2022-02-28	Hospital Universitario de Jaén (Jaén)	SAMEA110395613
AND21907	C	2022-03-08	Hospital Huércal-Overa (Almería)	SAMEA110395614
AND21955	C	2022-03-03	Hospital Universitario Virgen de la Victoria (Málaga)	SAMEA110395615
AND21956	C	2022-03-03	Hospital Universitario Virgen de la Victoria (Málaga)	SAMEA110395616
AND22019	C	2022-03-10	Hospital Universitario de Jaén (Jaén)	SAMEA110395617
AND22023	C	2022-03-09	Hospital Universitario de Jaén (Jaén)	SAMEA110395618
AND22043	R	2022-03-18	Hospital Universitario Virgen del Rocío (Sevilla)	SAMEA110395619
AND22078	C	2022-03-23	Hospital Universitario Virgen del Rocío (Sevilla)	SAMEA110395620

AND22272	C	2022-03-30	Hospital Universitario Virgen del Rocío (Sevilla)	SAMEA110395621
AND22275	C	2022-03-21	Hospital Universitario Juan Ramón Jiménez (Huelva)	SAMEA110395622
AND22431	C	2022-03-28	Hospital Universitario San Cecilio (Granada)	SAMEA110395623
AND22527	C	2022-03-14	Hospital Universitario Virgen de la Victoria (Málaga)	SAMEA110395624
AND22544	C	2022-03-17	Hospital Universitario Virgen de la Victoria (Málaga)	SAMEA110395625
AND22788	C	2022-04-07	Hospital Universitario San Cecilio (Granada)	SAMEA110395626
AND22794	C	2022-03-31	Hospital Universitario San Cecilio (Granada)	SAMEA110395627
AND22816	C	2022-03-31	Plataforma Provincial de Almería (Almería)	SAMEA110395628
AND22894	C	2022-03-21	Hospital Universitario Virgen de la Victoria (Málaga)	SAMEA110395629
AND22925	C	2022-03-21	Hospital Universitario Virgen de la Victoria (Málaga)	SAMEA110395630
AND22927	C	2022-03-28	Hospital Universitario Virgen de la Victoria (Málaga)	SAMEA110395631
AND22930	C	2022-04-05	Hospital Universitario Virgen de la Victoria (Málaga)	SAMEA110395632
AND23055*	R	2022-03-02	Hospital Universitario Reina Sofía (Córdoba)	SAMEA110395673
AND23187*	R	2022-02-18	Hospital Universitario Reina Sofía (Córdoba)	SAMEA110395633
AND23287	R	2022-04-18	Hospital Universitario Juan Ramón Jiménez (Huelva)	SAMEA110395634
AND23305	R	2022-04-20	Hospital Universitario Virgen del Rocío (Sevilla)	SAMEA110395635
AND23397	C	2022-04-25	Hospital Universitario San Cecilio (Granada)	SAMEA110395636
AND23422	C	2022-04-25	Hospital Universitario San Cecilio (Granada)	SAMEA110395637
AND23426	C	2022-04-25	Hospital Universitario San Cecilio (Granada)	SAMEA110395638
AND23508	C	2022-04-11	Hospital Universitario Virgen de la Victoria (Málaga)	SAMEA110395639
AND23521	R	2022-04-11	Hospital Universitario Virgen de la Victoria (Málaga)	SAMEA110395640
AND23565	C	2022-04-17	Hospital Universitario de Jaén (Jaén)	SAMEA110395641
AND23566	C	2022-04-18	Hospital Universitario de Jaén (Jaén)	SAMEA110395642
AND23569	C	2022-04-12	Hospital Universitario Poniente (El Ejido, Almería)	SAMEA110395643
AND23570	C	2022-04-07	Hospital Universitario Poniente (El Ejido, Almería)	SAMEA110395644
AND23576	C	2022-04-17	Hospital Universitario de Jaén (Jaén)	SAMEA110395645
AND23594	C	2022-04-12	Hospital Universitario de Jaén (Jaén)	SAMEA110395646

AND23635	R	2022-04-22	Hospital Universitario Virgen del Rocío (Sevilla)	SAMEA110395647
AND23728	R	2022-04-21	Hospital Universitario Juan Ramón Jiménez (Huelva)	SAMEA110395648
AND23732	R	2022-04-20	Hospital Universitario Juan Ramón Jiménez (Huelva)	SAMEA110395649
AND23734	R	2022-04-21	Hospital Universitario Juan Ramón Jiménez (Huelva)	SAMEA110395650
AND23786	R	2022-04-19	Hospital Universitario Juan Ramón Jiménez (Huelva)	SAMEA110395651
AND23787	R	2022-04-19	Hospital Universitario Juan Ramón Jiménez (Huelva)	SAMEA110395652
AND23791	R	2022-04-25	Hospital Universitario Juan Ramón Jiménez (Huelva)	SAMEA110395653
AND23912	C	2022-04-22	Hospital Universitario de Jaén (Jaén)	SAMEA110395654
AND24108	C	2022-04-26	Hospital Universitario Virgen de la Victoria (Málaga)	SAMEA110395655
AND24298	R	2022-05-07	Hospital Universitario Juan Ramón Jiménez (Huelva)	SAMEA110395656
AND24301	R	2022-04-27	Hospital Universitario Juan Ramón Jiménez (Huelva)	SAMEA110395657
AND24302	R	2022-04-27	Hospital Universitario Juan Ramón Jiménez (Huelva)	SAMEA110395658
AND24350	R	2022-05-12	Hospital Universitario Virgen del Rocío (Sevilla)	SAMEA110395659
AND24357	R	2022-05-12	Hospital Universitario Virgen del Rocío (Sevilla)	SAMEA110395660
AND24465	C	2022-05-10	Hospital Universitario San Cecilio (Granada)	SAMEA110395661
AND24480	C	2022-05-11	Hospital Universitario San Cecilio (Granada)	SAMEA110395662
AND24658	R	2022-05-10	Hospital Universitario de Jaén (Jaén)	SAMEA110395663
AND24834	R	2022-05-16	Hospital Universitario Juan Ramón Jiménez (Huelva)	SAMEA110395664
AND24843	R	2022-05-16	Hospital Universitario Juan Ramón Jiménez (Huelva)	SAMEA110395665
AND24864	R	2022-05-09	Hospital Universitario Juan Ramón Jiménez (Huelva)	SAMEA110395666
AND24884	R	2022-05-18	Hospital Universitario Juan Ramón Jiménez (Huelva)	SAMEA110395667
AND25120	C	2022-05-17	Hospital Universitario San Cecilio (Granada)	SAMEA110395668
AND25238	C	2022-05-16	Hospital Universitario de Jaén (Jaén)	SAMEA110395669
AND25239	C	2022-05-21	Hospital Universitario de Jaén (Jaén)	SAMEA110395670
AND25258	R	2022-05-10	Hospital Universitario Virgen de la Victoria (Málaga)	SAMEA110395671
AND25339	C	2022-05-16	Hospital Universitario de Jaén (Jaén)	SAMEA110395672

* Samples correspond to the same patient

Table S2. SARS-CoV-2 sequences compatible with co-infections

Sample	Clade / nextclade pango (Nextclade)	Lineage (Pangolin)	#Breakpoints (sc2rf)	Lollipop plot
AND17788	21K / BA.1	Unassigned	8	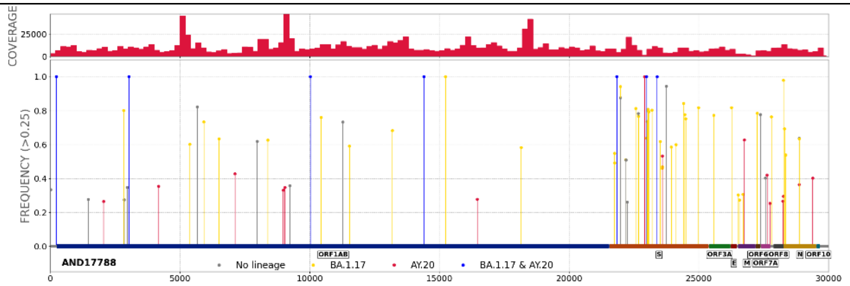 Delta – Omicron coinfection
AND17799	21K / BA.1.17	Unassigned	8	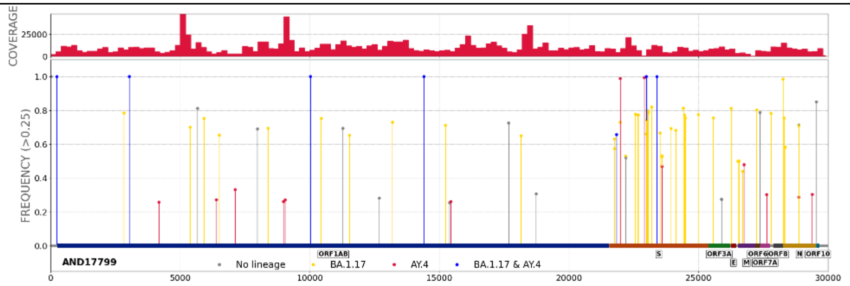 Delta – Omicron coinfection
AND19065	20B / B.1.1	Unassigned	4	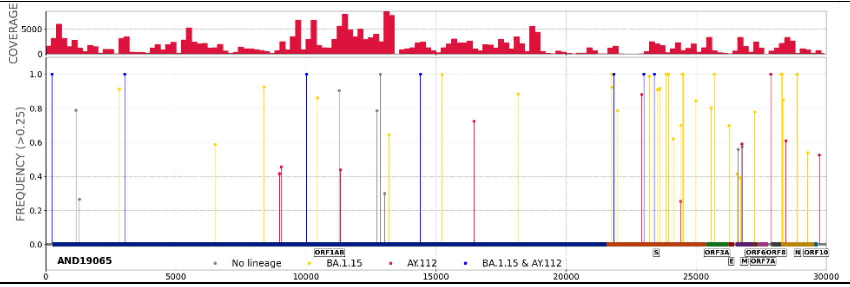

				Delta – Omicron coinfection
AND19148	20B / B.1.1	Unassigned	18	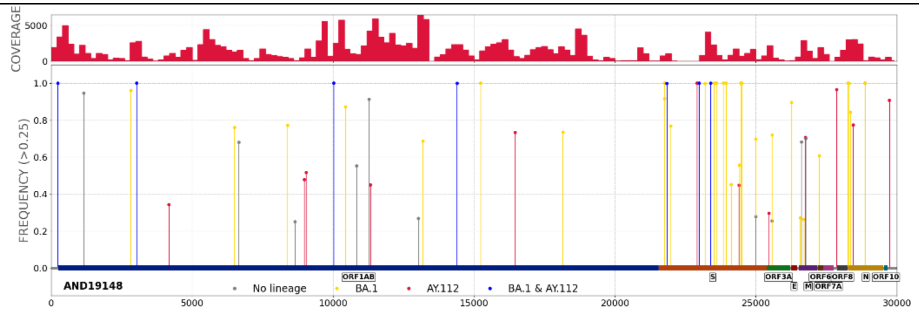 Delta-Omicron coinfection
AND19238	20B / B.1.1	Unassigned	16	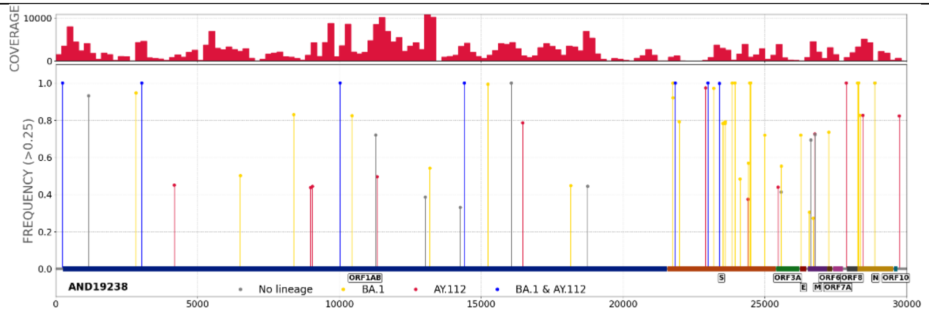 Delta-Omicron coinfection

AND19468	20B / B.1.1	Unassigned	12	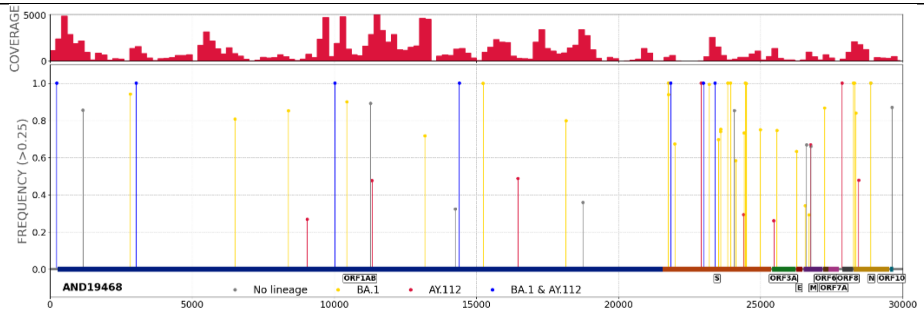<br>Delta-Omicron coinfection
AND19571	20B / B.1.1	Unassigned	20	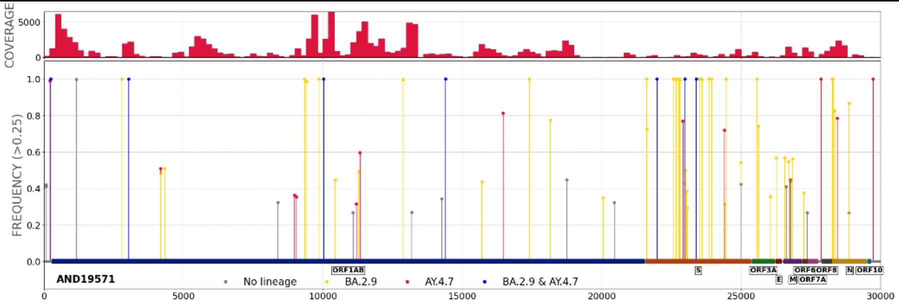<br>Delta-Omicron coinfection
AND20157	B.1.1.529	Unassigned	-	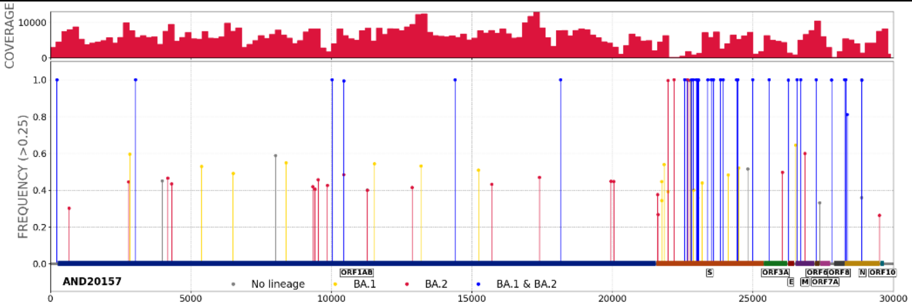<br>Omicron BA.1 – Omicron BA.2 coinfection

AND20304	21M / B.1.1.529	Unassigned	4	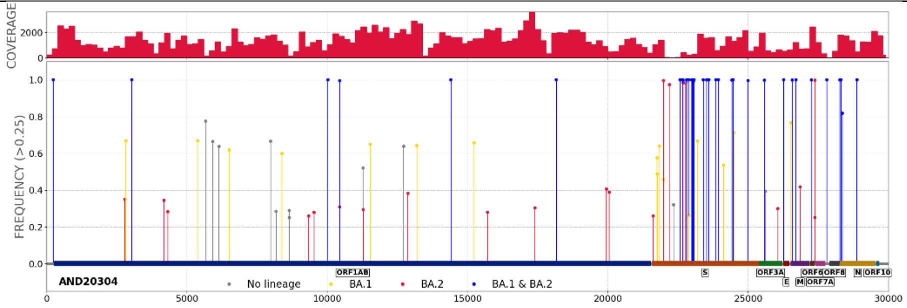<br>Omicron BA.1 – Omicron BA.2 coinfection
AND20601	21M / B.1.1.529	Unassigned	-	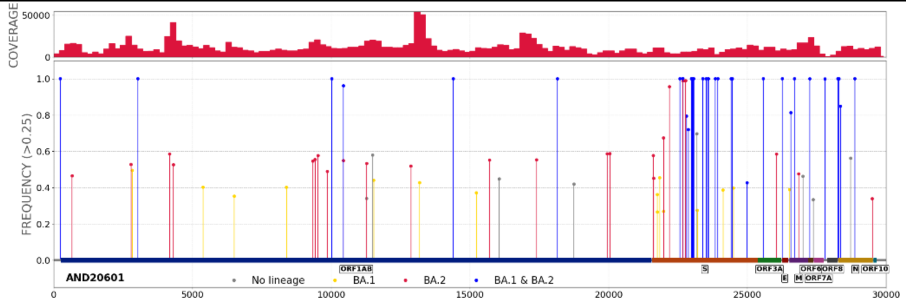<br>Omicron BA.1 – Omicron BA.2 coinfection
AND20905	recombinant / XT (BA.1-BA.2)	Unassigned	-	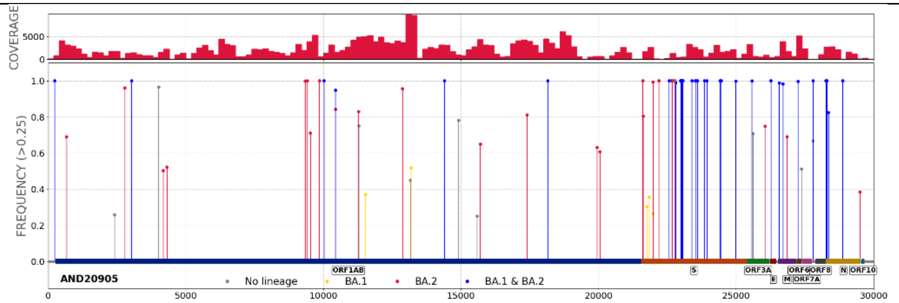<br>Omicron BA.1 – Omicron BA.2 coinfection

<table>
<tr><td>AND20906</td><td>recombinant / XT (BA.1-BA.2)</td><td>Unassigned</td><td>9</td><td>
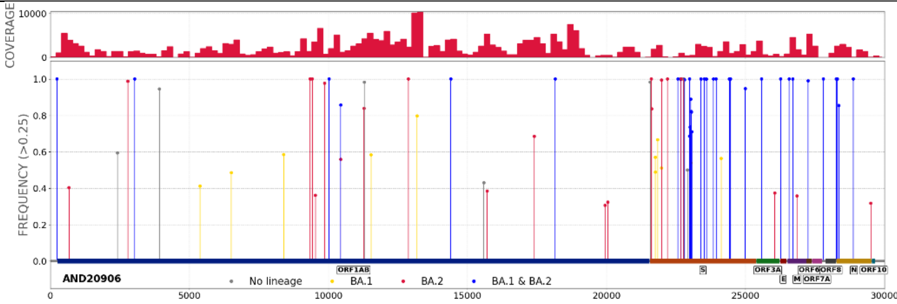

Omicron BA.1 – Omicron BA.2 coinfection</td></tr>
<tr><td>AND20966</td><td>recombinant / XT (BA.1-BA.2)</td><td>Unassigned</td><td>9</td><td>
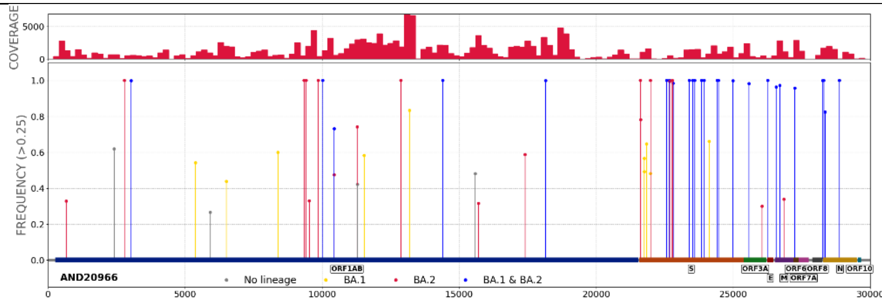

Omicron BA.1 – Omicron BA.2 coinfection</td></tr>
<tr><td>AND21001</td><td>recombinant / XT (BA.1-BA.2)</td><td>Unassigned</td><td>-</td><td>
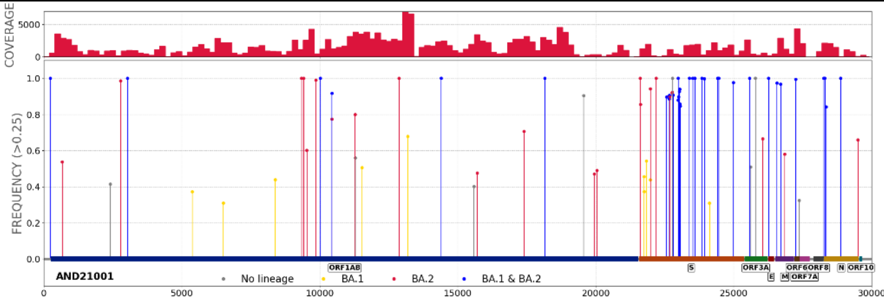

Omicron BA.1 – Omicron BA.2 coinfection</td></tr>
</table>

AND21137	21L / BA.2.23	Unassigned	-	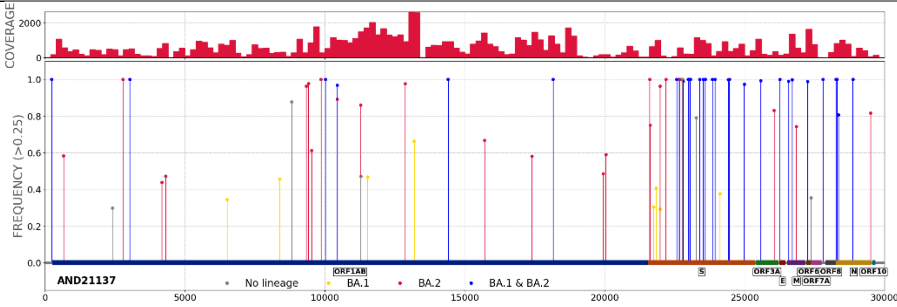<br>Omicron BA.1 – Omicron BA.2 coinfection
AND21375	21M/ B.1.1.529	Unassigned	1	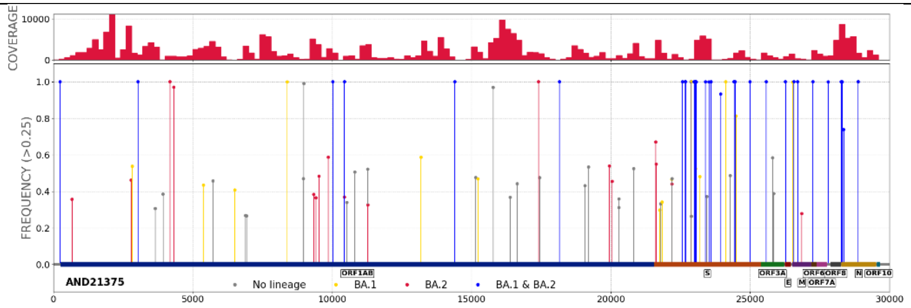<br>Omicron BA.1 – Omicron BA.2 coinfection
AND21391	21L / BA.2.23	Unassigned	-	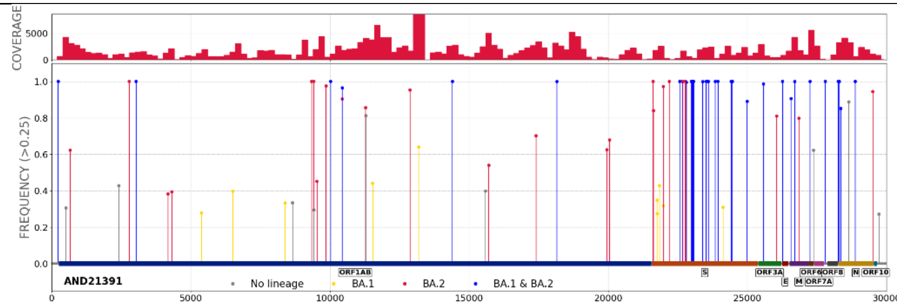<br>Omicron BA.1 – Omicron BA.2 coinfection

AND21405	recombinant / XT (BA.1-BA.2)	Unassigned	-	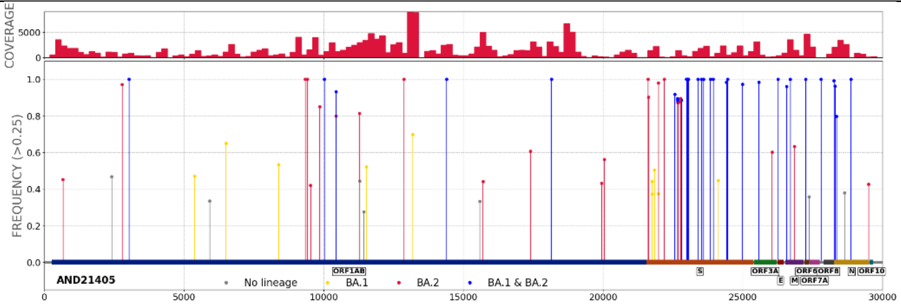 Omicron BA.1 – Omicron BA.2 coinfection
AND21422	21L / BA.2.23	Unassigned	-	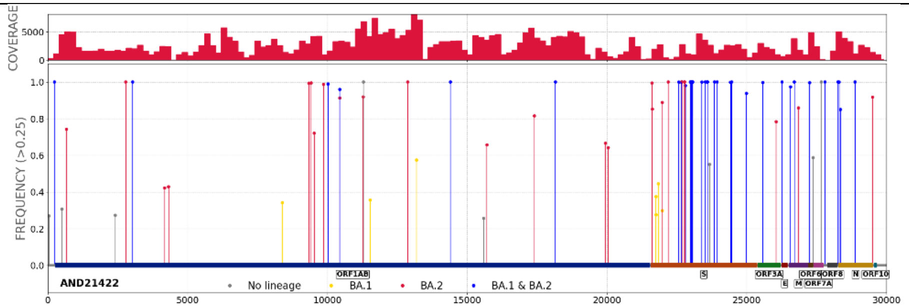 Omicron BA.1 – Omicron BA.2 coinfection
AND21474	recombinant / XT (BA.1-BA.2)	Unassigned	7	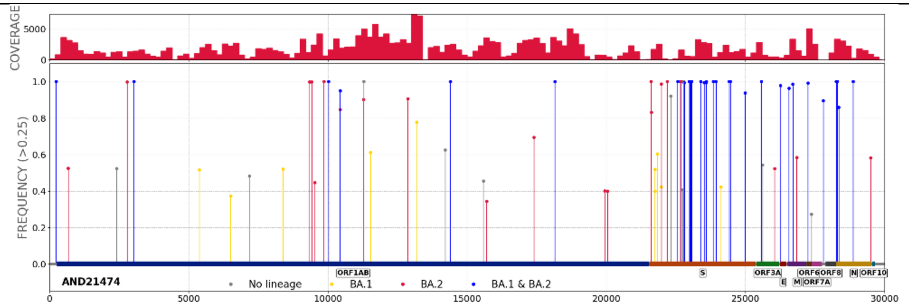 Omicron BA.1 – Omicron BA.2 coinfection

<table>
<tr><td>AND21475</td><td>21K / BA.1</td><td>Unassigned</td><td>2</td><td>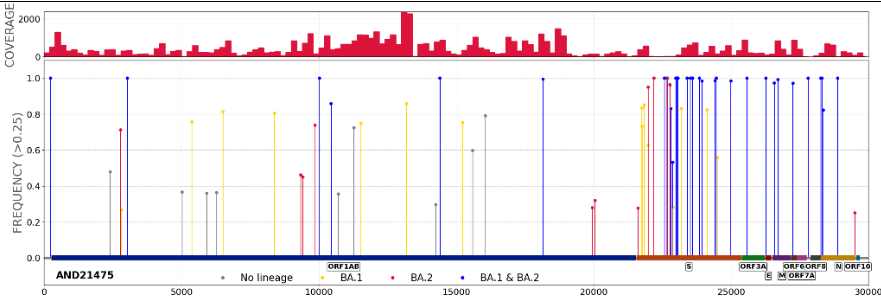
Omicron BA.1 – Omicron BA.2 coinfection</td></tr>
<tr><td>AND21483</td><td>recombinant / XT (BA.1-BA.2)</td><td>Unassigned</td><td>18</td><td>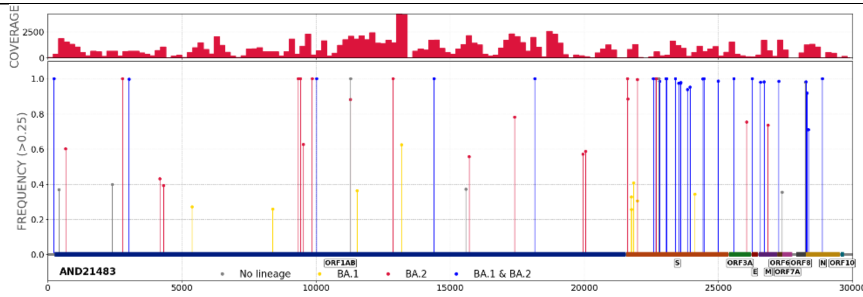
Omicron BA.1 – Omicron BA.2 coinfection</td></tr>
<tr><td>AND21520</td><td>21M / B.1.1.529</td><td>Unassigned</td><td>14</td><td>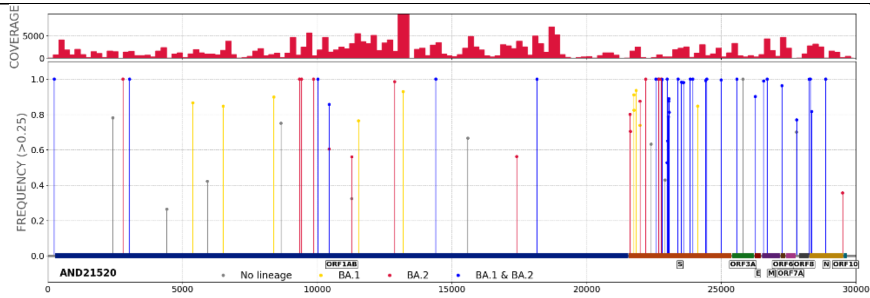
Omicron BA.1 – Omicron BA.2 coinfection</td></tr>
</table>

AND21550	21L / BA.2.23	Unassigned	-	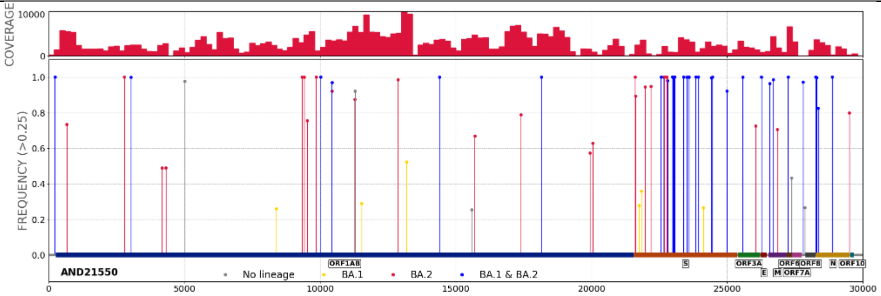<br>Omicron BA.1 – Omicron BA.2 coinfection
AND21552	recombinant / XT (BA.1-BA.2)	Unassigned	9	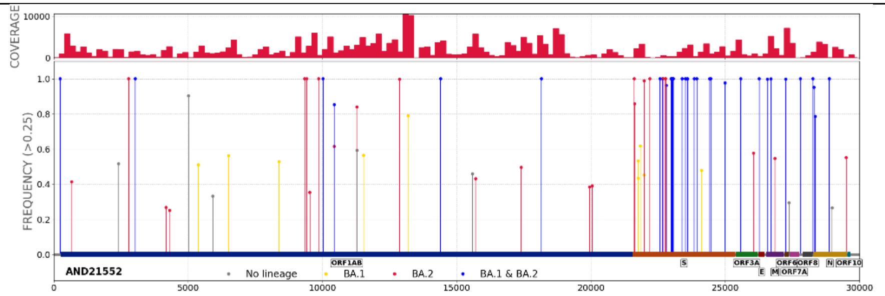<br>Omicron BA.1 – Omicron BA.2 coinfection
AND21554	recombinant / XT (BA.1-BA.2)	Unassigned	-	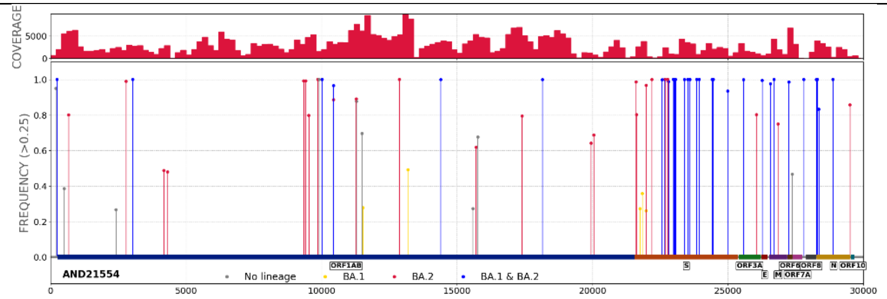<br>Omicron BA.1 – Omicron BA.2 coinfection

<table>
<tr><td>AND21702</td><td>21M / B.1.1.529</td><td>Unassigned</td><td>-</td><td>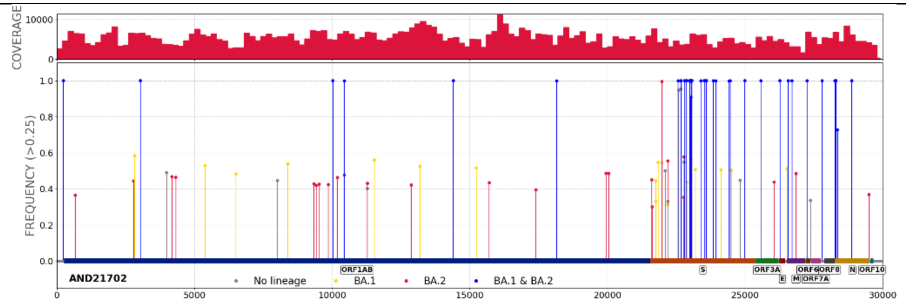

Omicron BA.1 – Omicron BA.2 coinfection</td></tr>
<tr><td>AND21845</td><td>21L / BA.2</td><td>Unassigned</td><td>-</td><td>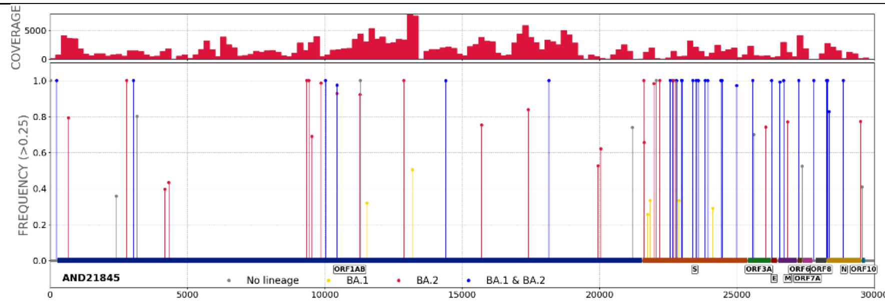

Omicron BA.1 – Omicron BA.2 coinfection</td></tr>
<tr><td>AND21903</td><td>21K / BA.1.1</td><td>Unassigned</td><td>4</td><td>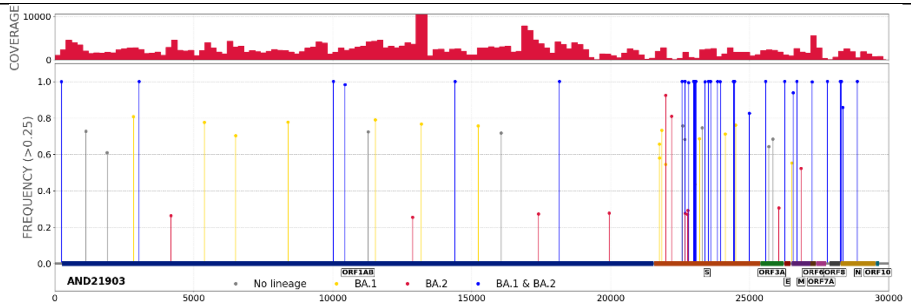

Omicron BA.1 – Omicron BA.2 coinfection</td></tr>
</table>

<table>
<tr><th></th><th></th><th></th><th></th><th></th></tr>
<tr><td>AND21907</td><td>recombinant / XT (BA.1-BA.2)</td><td>Unassigned</td><td>1</td><td>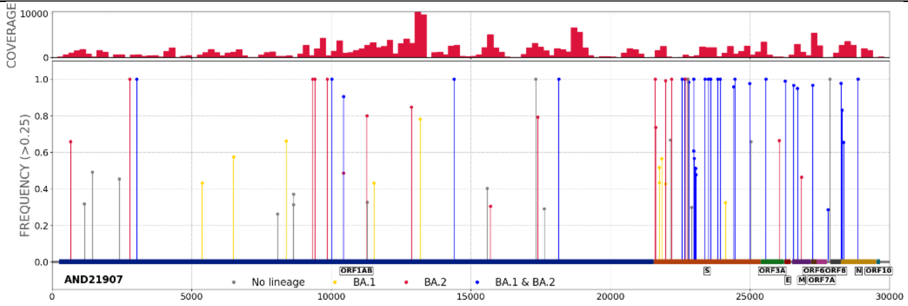

Omicron BA.1 – Omicron BA.2 coinfection</td></tr>
<tr><td>AND21955</td><td>21L / BA.2.23</td><td>Unassigned</td><td>8</td><td>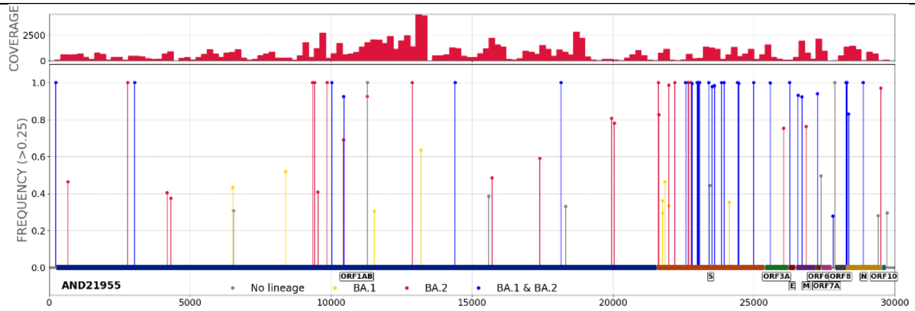

Omicron BA.1 – Omicron BA.2 coinfection</td></tr>
<tr><td>AND21956</td><td>recombinant / XT (BA.1-BA.2)</td><td>Unassigned</td><td>20</td><td>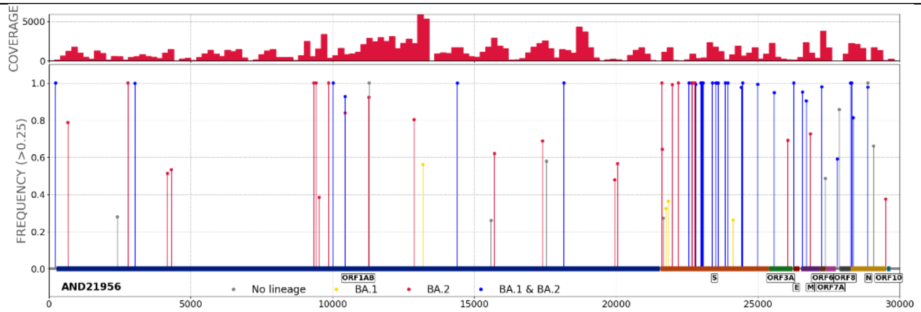

Omicron BA.1 – Omicron BA.2 coinfection</td></tr>
</table>

AND22019	recombinant / XT (BA.1-BA.2)	Unassigned	-	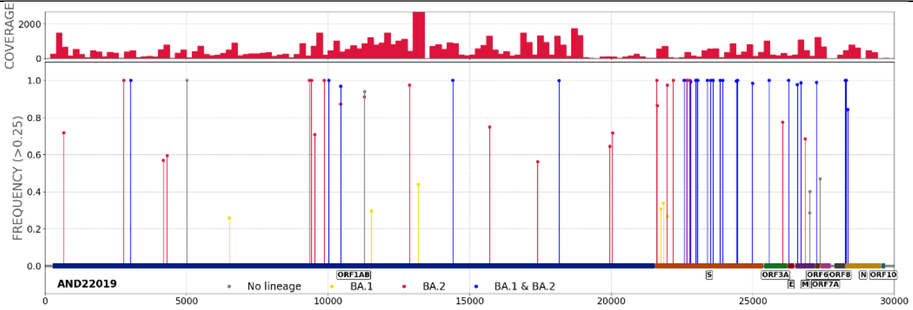 Omicron BA.1 – Omicron BA.2 coinfection
AND22023	21L / BA.2.23	Unassigned	-	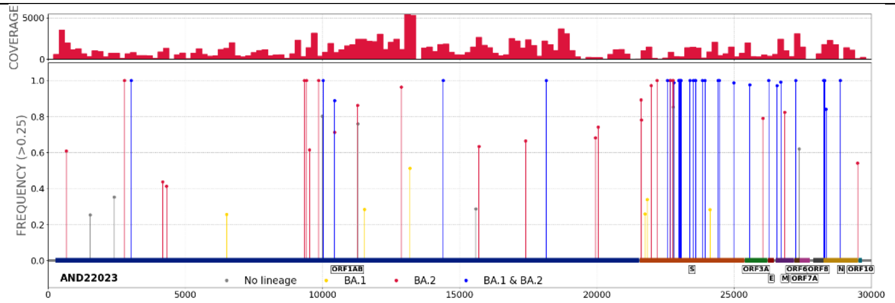 Omicron BA.1 – Omicron BA.2 coinfection
AND22078	21M / B.1.1.529	Unassigned	-	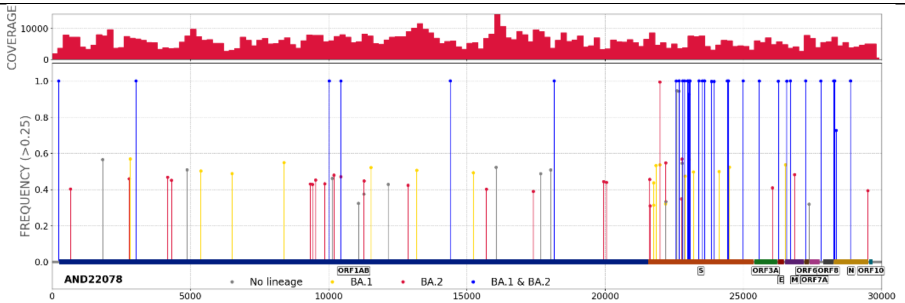 Omicron BA.1 – Omicron BA.2 coinfection

AND22272	21M / B.1.1.529	Unassigned	-	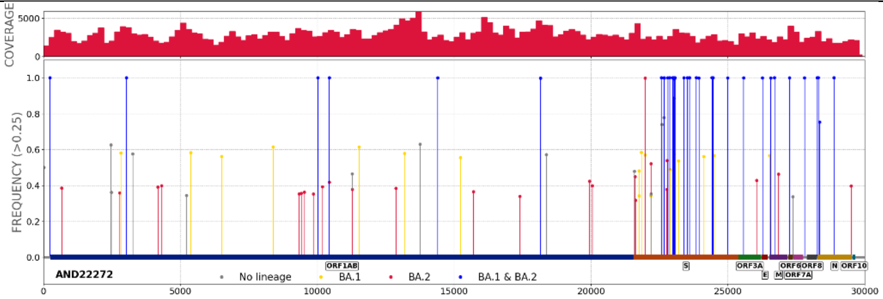<br>Omicron BA.1 – Omicron BA.2 coinfection
AND22275	21M / B.1.1.529	Unassigned	-	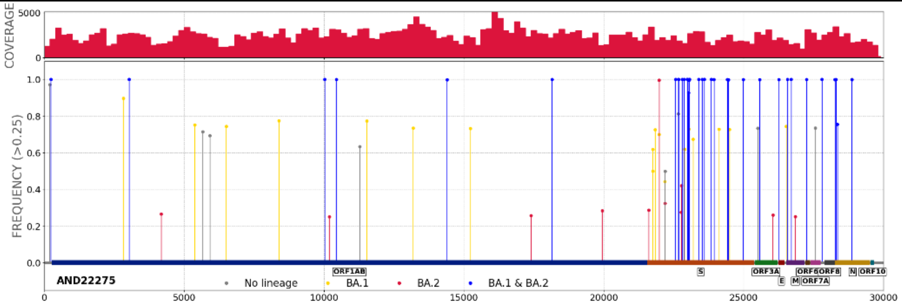<br>Omicron BA.1 – Omicron BA.2 coinfection
AND22431	21L / BA.2.23	Unassigned	9	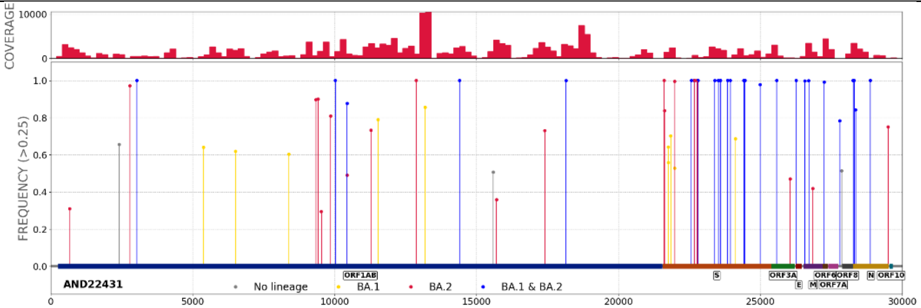<br>Omicron BA.1 – Omicron BA.2 coinfection

AND22527	21L / BA.2.23	Unassigned	18	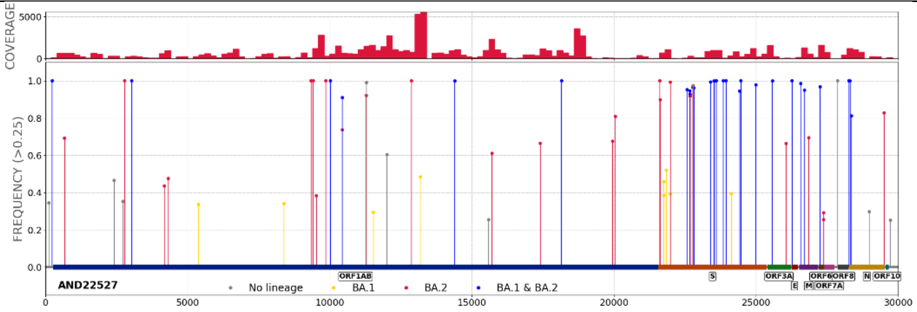 Omicron BA.1 – Omicron BA.2 coinfection
AND22544	recombinant / XT (BA.1-BA.2)	Unassigned	18	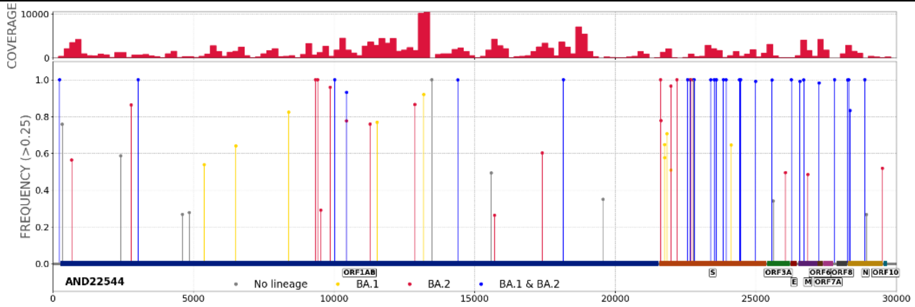 Omicron BA.1 – Omicron BA.2 coinfection
AND22788	recombinant / XT (BA.1-BA.2)	Unassigned	10	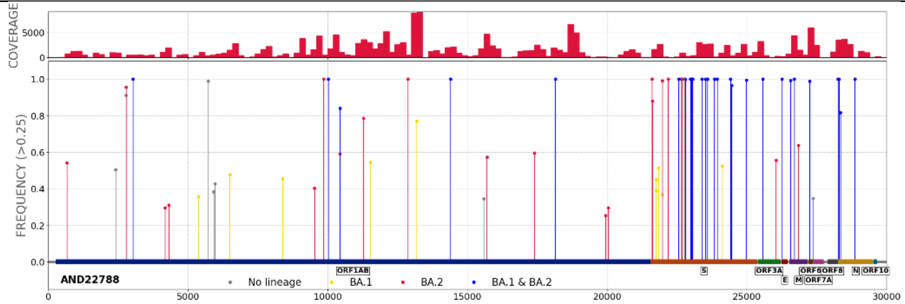 Omicron BA.1 – Omicron BA.2 coinfection

<table>
<tr><td>AND22794</td><td>recombinant / XT
(BA.1-BA.2)</td><td>Unassigned</td><td>9</td><td>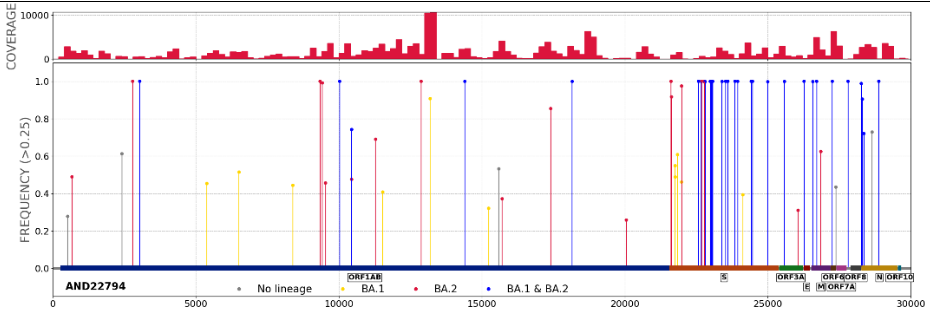

Omicron BA.1 – Omicron BA.2 coinfection</td></tr>
<tr><td>AND22816</td><td>21M / B.1.1.529</td><td>Unassigned</td><td>-</td><td>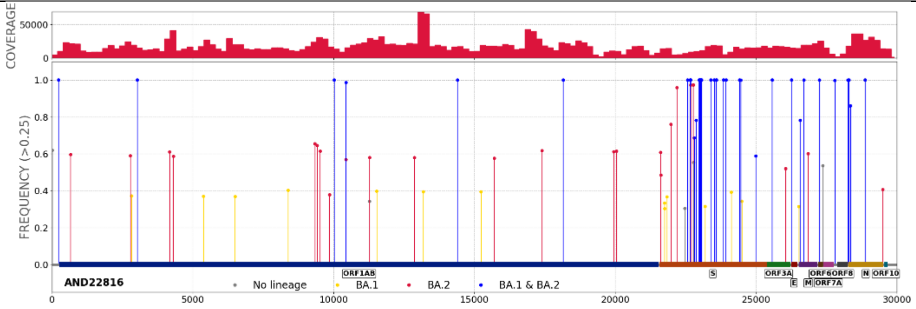

Omicron BA.1 – Omicron BA.2 coinfection</td></tr>
<tr><td>AND22894</td><td>recombinant / XT
(BA.1-BA.2)</td><td>Unassigned</td><td>7</td><td>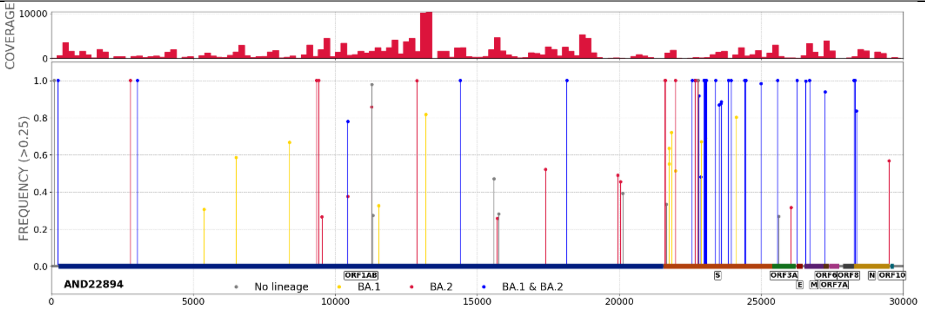
</td></tr>
</table>

				Omicron BA.1 – Omicron BA.2 coinfection
AND22925	recombinant / XT (BA.1-BA.2)	Unassigned	-	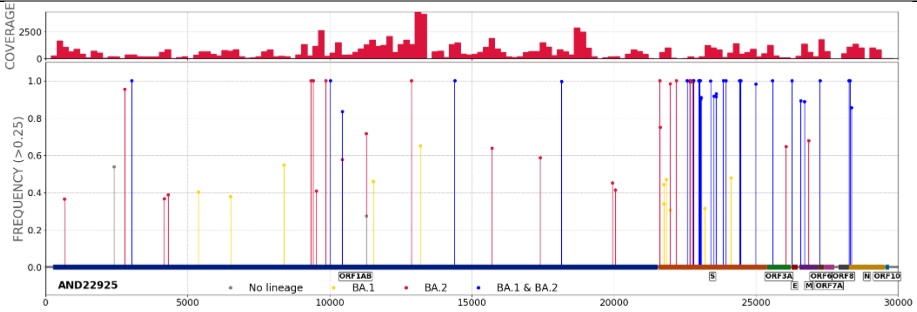
AND22927	21L / BA.2.23	Unassigned	-	Omicron BA.1 – Omicron BA.2 coinfection<br>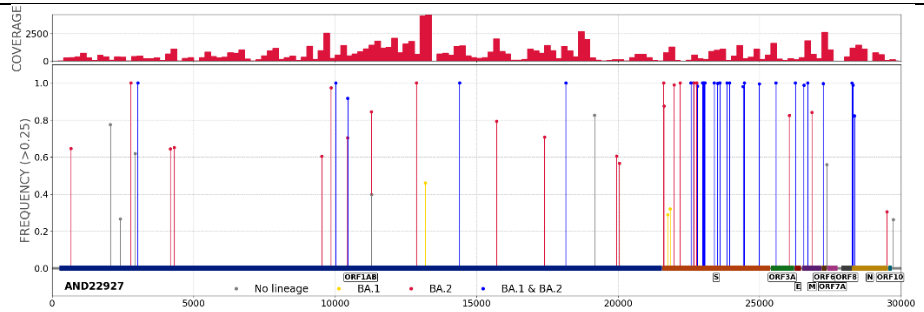
AND22930	recombinant / XT (BA.1-BA.2)	Unassigned	11	Omicron BA.1 – Omicron BA.2 coinfection<br>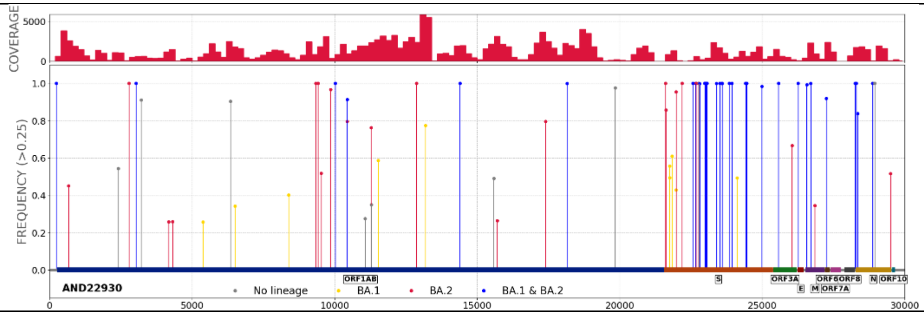

				Omicron BA.1 – Omicron BA.2 coinfection
AND23397	21L / BA.2.23	Unassigned	1	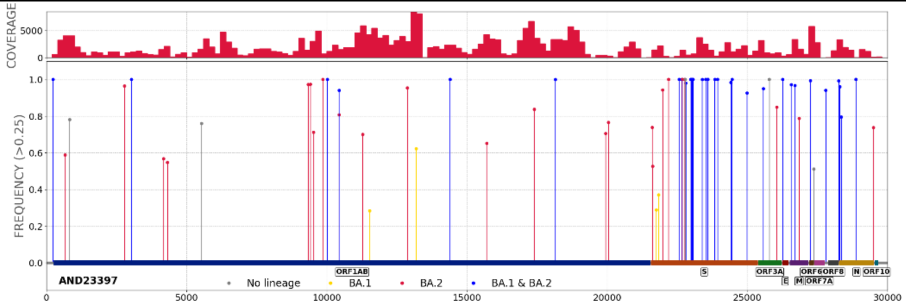<br>Omicron BA.1 – Omicron BA.2 coinfection
AND23422	21L / BA.2.23	Unassigned	8	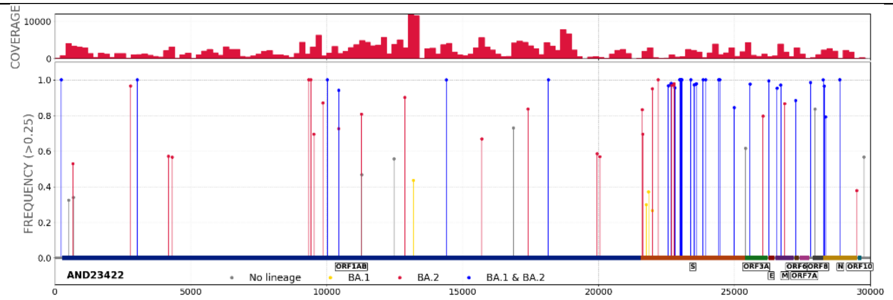<br>Omicron BA.1 – Omicron BA.2 coinfection
AND23426	recombinant / XT (BA.1-BA.2)	Unassigned	-	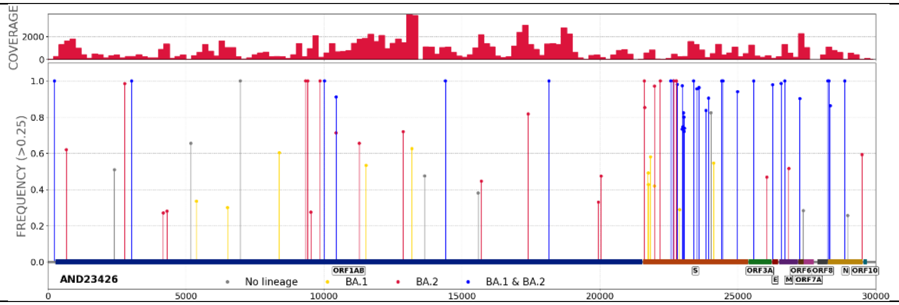

				Omicron BA.1 – Omicron BA.2 coinfection
AND23508	recombinant / XQ (BA.1.1-BA.2)	Unassigned	-	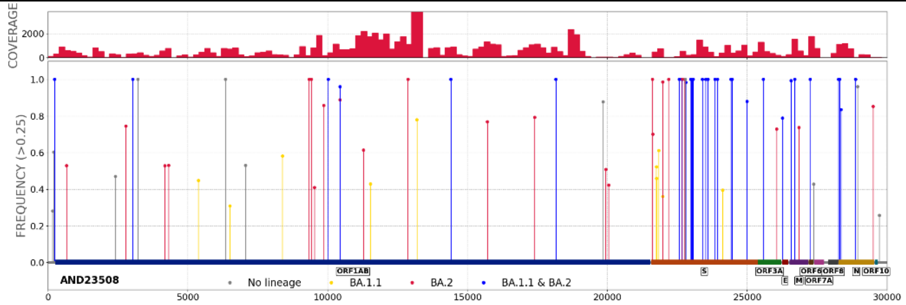<br>Omicron BA.1 – Omicron BA.2 coinfection
AND23565	21M / B.1.1.529	Unassigned	26	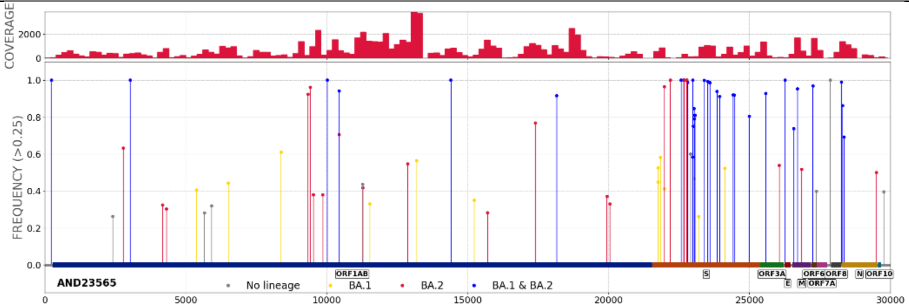<br>Omicron BA.1 – Omicron BA.2 coinfection
AND23566	21M / B.1.1.529	Unassigned	-	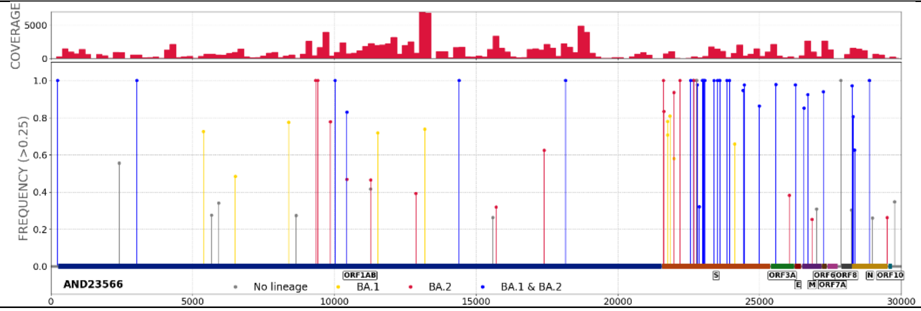

				Omicron BA.1 – Omicron BA.2 coinfection
AND23569	21M /B.1.1.529	Unassigned	2	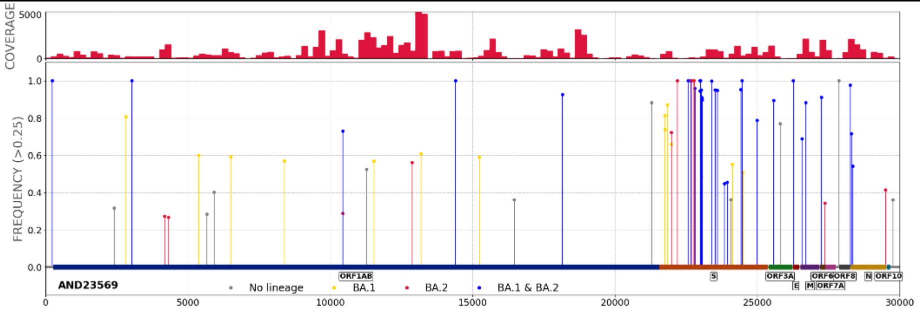 Omicron BA.1 – Omicron BA.2 coinfection
AND23570	21M /B.1.1.529	Unassigned	-	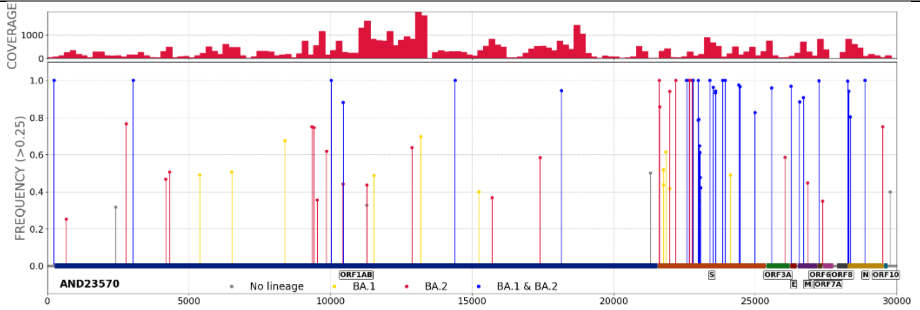 Omicron BA.1 – Omicron BA.2 coinfection
AND23576	21M / B.1.1.529	Unassigned	-	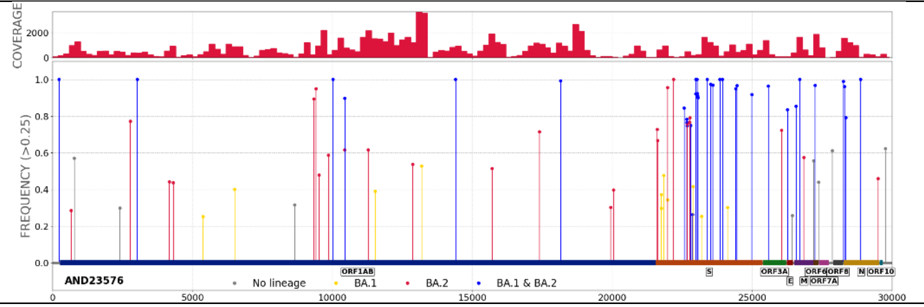

				Omicron BA.1 – Omicron BA.2 coinfection
AND23594	21M / B.1.1.529	Unassigned	-	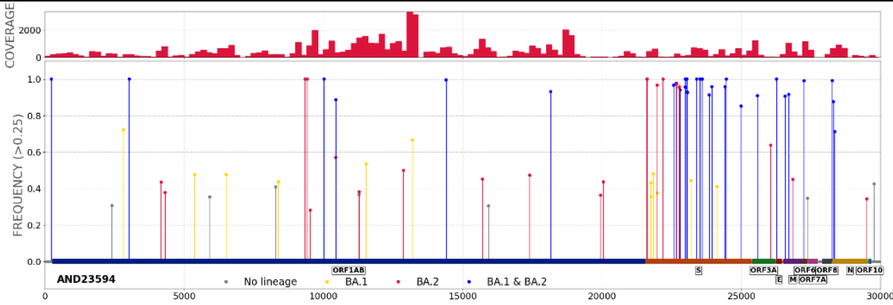<br>Omicron BA.1 – Omicron BA.2 coinfection
AND23912	21M / B.1.1.529	Unassigned	4	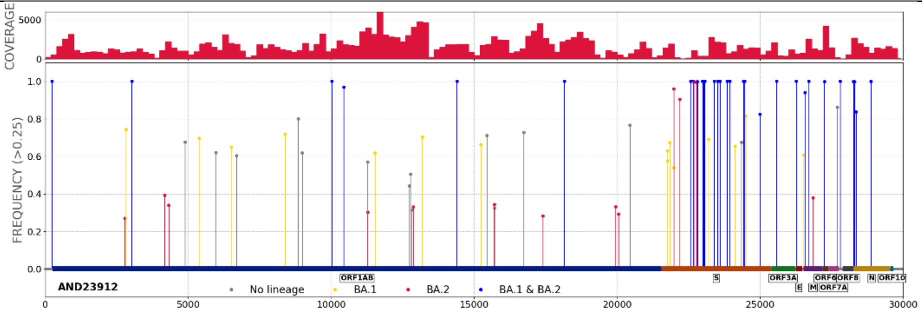<br>Omicron BA.1 – Omicron BA.2 coinfection
AND24108	recombinant / XT (BA.1-BA.2)	Unassigned	-	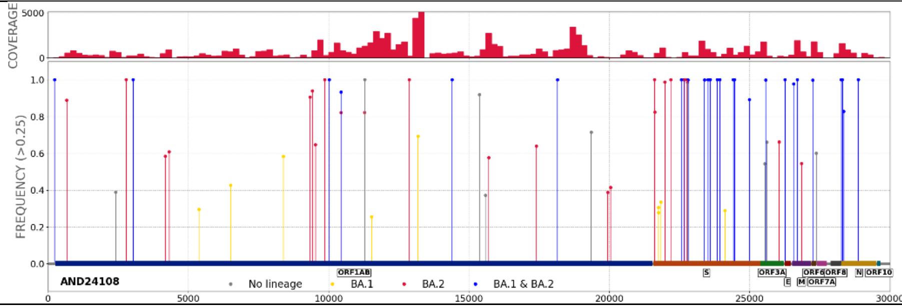

AND24465	21M / B.1.1.529	Unassigned	-	Omicron BA.1 – Omicron BA.2 coinfection<br>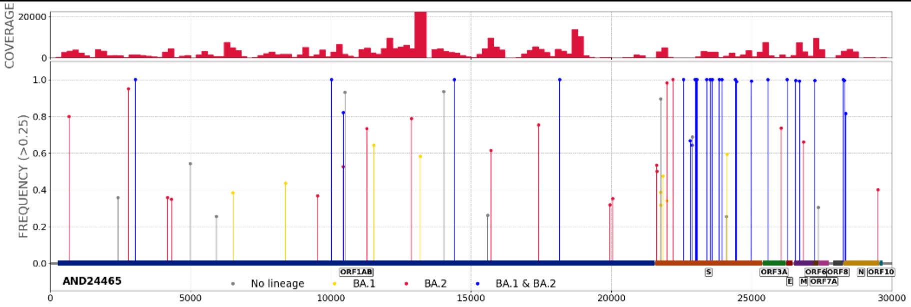
AND24480	recombinant / XT (BA.1-BA.2)	Unassigned	-	Omicron BA.1 – Omicron BA.2 coinfection<br>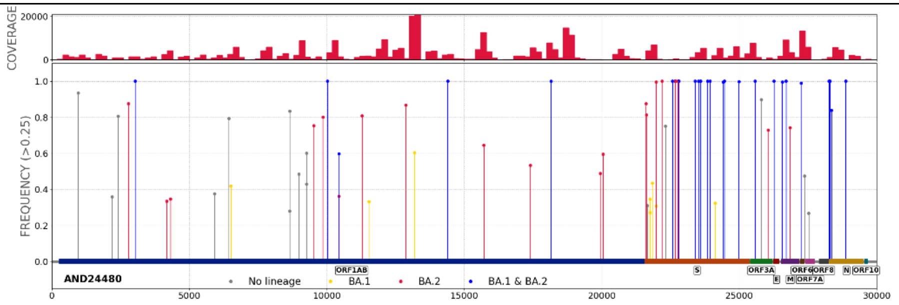
AND25120	21M / B.1.1.529	Unassigned	1	Omicron BA.1 – Omicron BA.2 coinfection<br>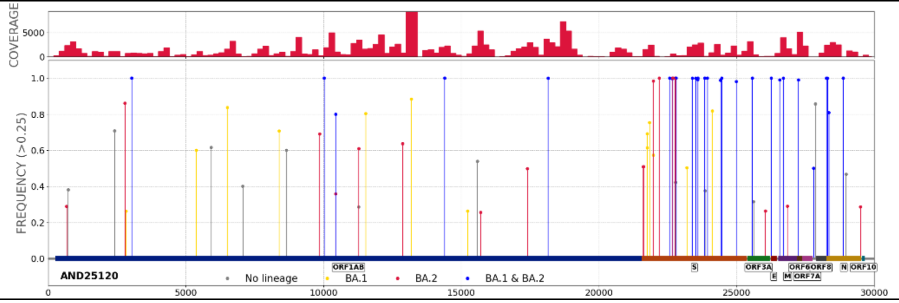

AND25238	recombinant / XT (BA.1-BA.2)	Unassigned	-	Omicron BA.1 – Omicron BA.2 coinfection 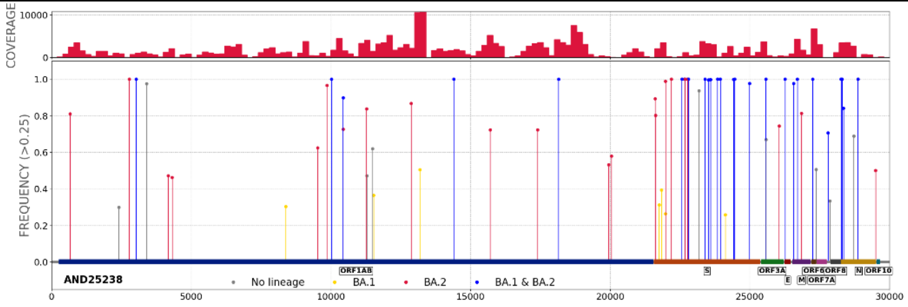
AND25239	21M / B.1.1.529	Unassigned	-	Omicron BA.1 – Omicron BA.2 coinfection 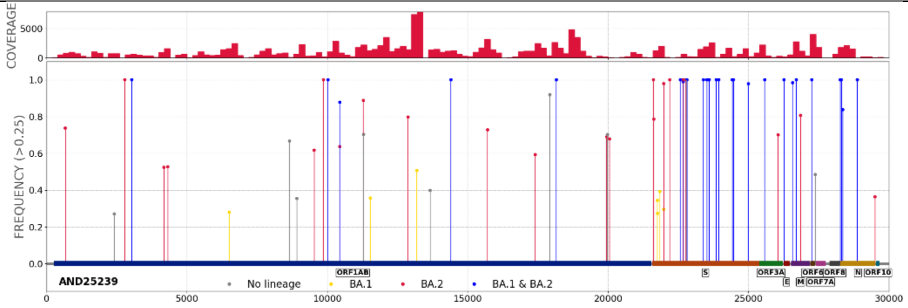
AND25339	recombinant / XT (BA.1-BA.2)	Unassigned	-	Omicron BA.1 – Omicron BA.2 coinfection 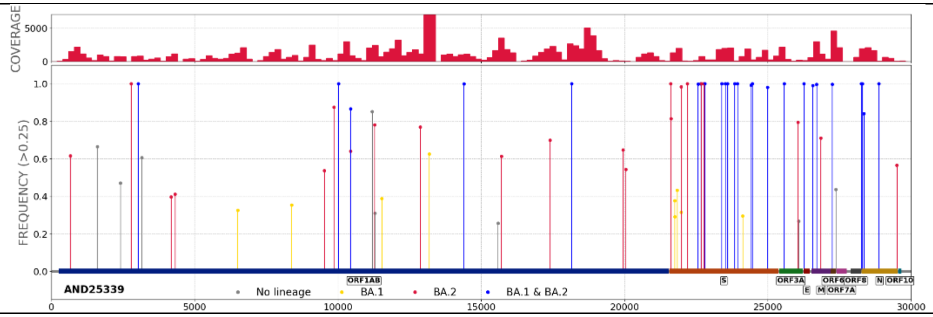

				Omicron BA.1 – Omicron BA.2 coinfection

Table S3. Potential SARS-CoV-2 recombinant

Sample	Clade / nextclade pango (Nextclade)	Lineage (Pangolin)	Breakpoints output (sc2rf)	Lollipop plot
AND21237*	recombinant / XD (AY.4-BA.1)	AY.93	10029-10449 (nsp5)<br><br>25000-25469 (S or ORF3a)	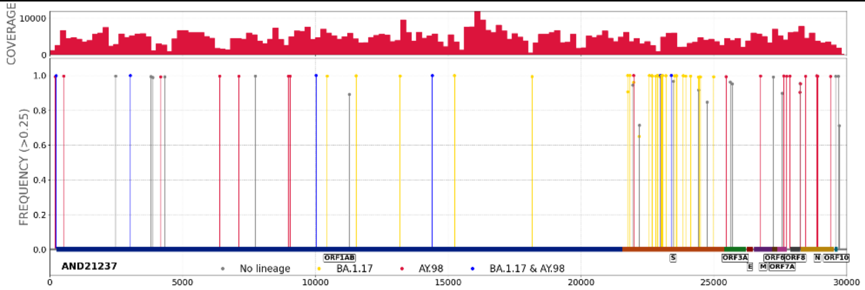<br><br>Novel recombinant Delta-Omicron-Delta (Illumina sequencing)
AND21266	recombinant / XM (BA.1.1-BA.2)	Unassigned	5386-8393 (nsp3)<br><br>20055-21617 (nsp15 or S)	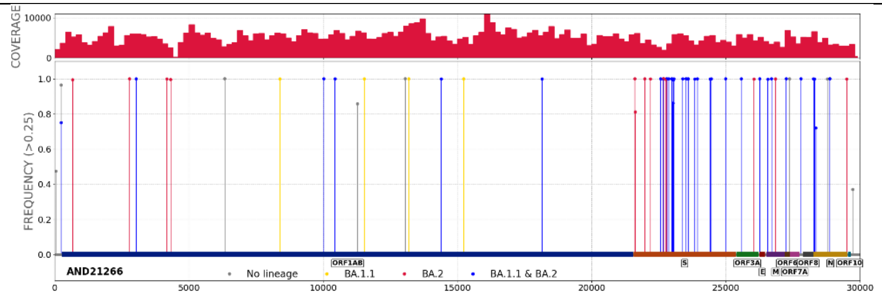

				Novel recombinante Omicron BA.2-BA.1-BA.2
AND21532	recombinant / XM (BA.1.1- BA.2)	Unassigned	20055-21618 (nsp15 or S)	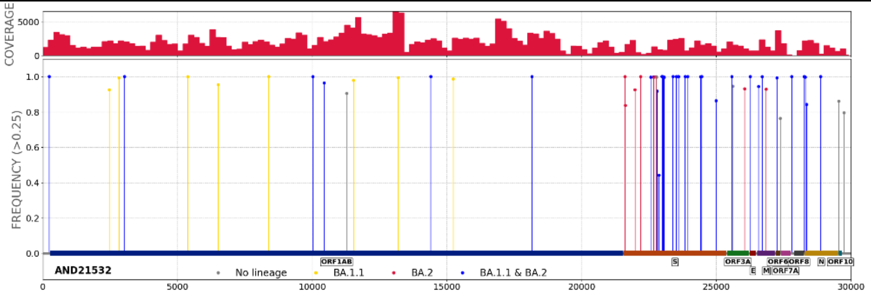 Breakpoint close to XM (17410-19995)
AND22043	recombinant / XM (BA.1.1- BA.2)	Unassigned	5386-8393 (nsp3)<br><br>20055-21618 (nsp15 or S)	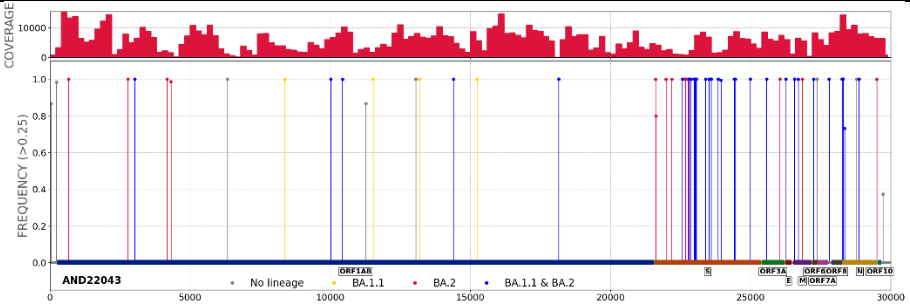 Novel recombinant Omicron BA.2-BA.1-BA.2

AND23055*	recombinant / XD (AY.4 – BA.1)	BA.1	10029-10449 (nsp5)<br><br>25000-25469 (S or ORF3a)	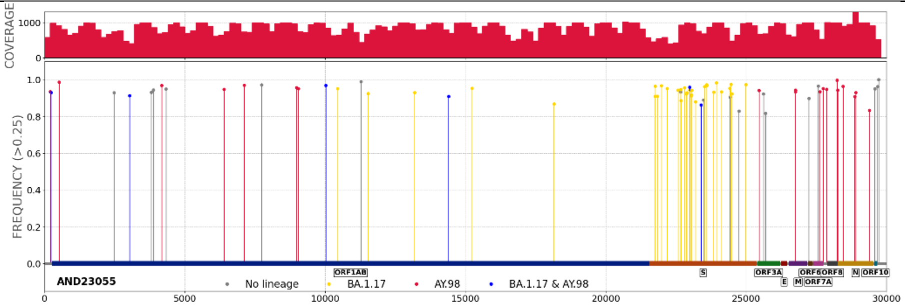<br>Novel recombinant Delta-Omicron-Delta (Nanopore sequencing)
AND23187*	recombinant / XD (AY.4 – BA.1)	BA.1	10029-10449 (nsp5)<br><br>25000-25469 (S or ORF3a)	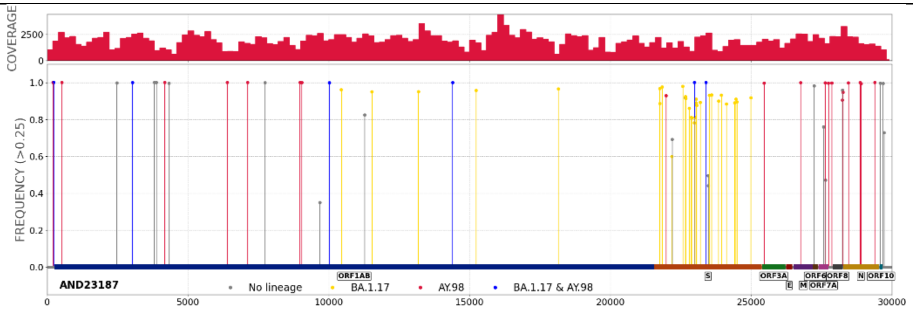<br>Novel recombinant Delta-Omicron-Delta (Illumina sequencing)

AND23287	recombinant / XQ (BA.1.1- BA.2)	BA.2	4184-4321 (nsp3)	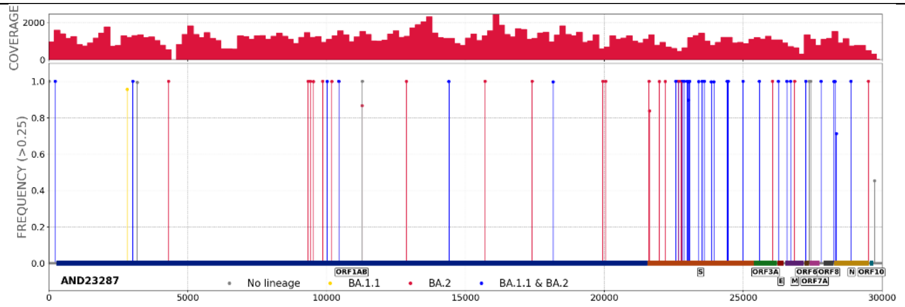<br>Breakpoint close to XQ (4322-5385) or XR (4322-4891) recombinant
AND23305	recombinant / XT (BA.1-BA.2)	BA.2	25584-26060 (S or ORF3a)	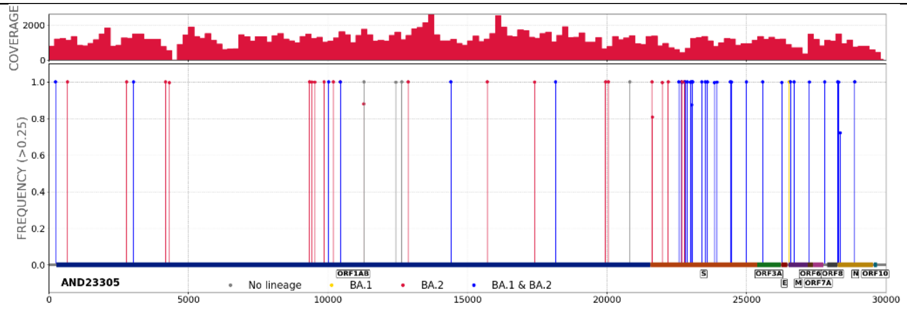<br>Breakpoint close to XT (26062-26528) and XAE (24506-26048)

AND23521	recombinant / XQ (BA.1.1- BA.2)	BA.2	4184-4321 (nsp3)	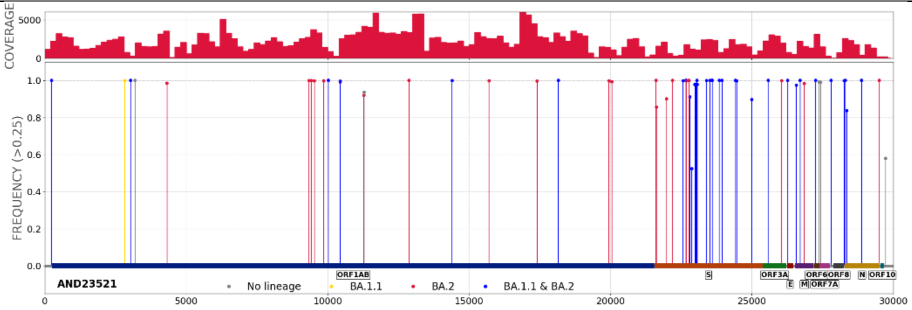<br>Breakpoint close to XQ (4322-5385) or XR (4322-4891) recombinant
AND23635	recombinant / XT (BA.1-BA.2)	BA.2	25584-26060 (S or ORF3a)	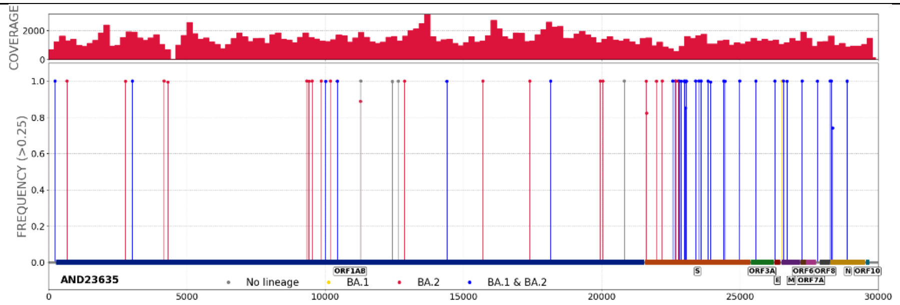<br>Breakpoint close to XT (26062-26528) and XAE (24506-26048)

AND23728	recombinant / XQ (BA.1.1-BA.2)	BA.2	4184-4321 (nsp3)	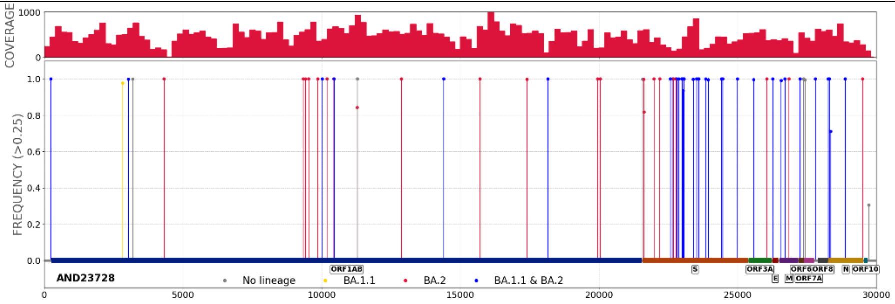<br>Breakpoint close to XQ (4322-5385) or XR (4322-4891) recombinant
AND23732	recombinant / XQ (BA.1.1-BA.2)	BA.2	4184-4321 (nsp3)	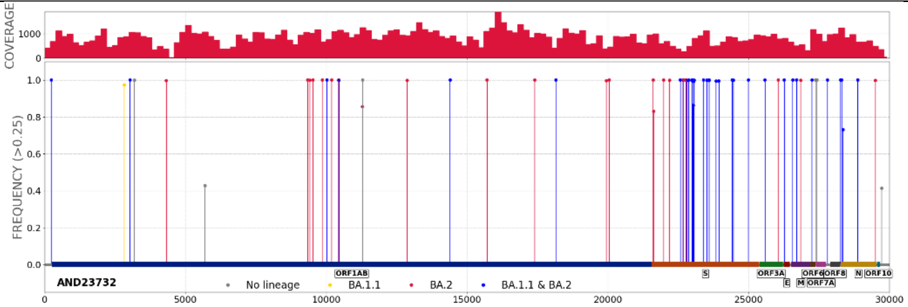<br>Breakpoint close to XQ (4322-5385) or XR (4322-4891) recombinant

<table>
<tr><td></td><td></td><td></td><td></td><td></td></tr>
<tr><td>AND23734</td><td>recombinant / XQ (BA.1.1-BA.2)</td><td>BA.2</td><td>4184-4321 (nsp3)</td><td>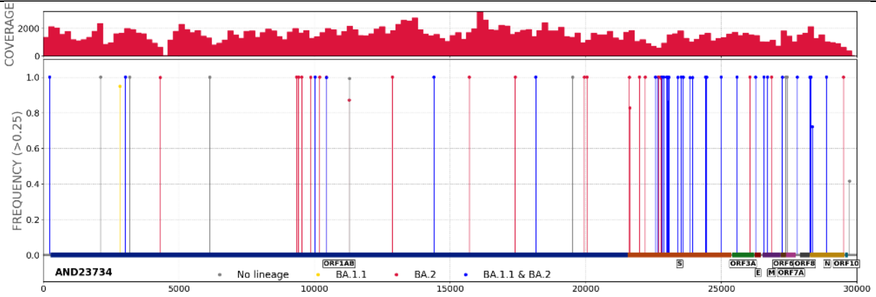

Breakpoint close to XQ (4322-5385) or XR (4322-4891) recombinant</td></tr>
<tr><td>AND23786</td><td>recombinant / XQ (BA.1.1-BA.2)</td><td>BA.2</td><td>4184-4321 (nsp3)</td><td>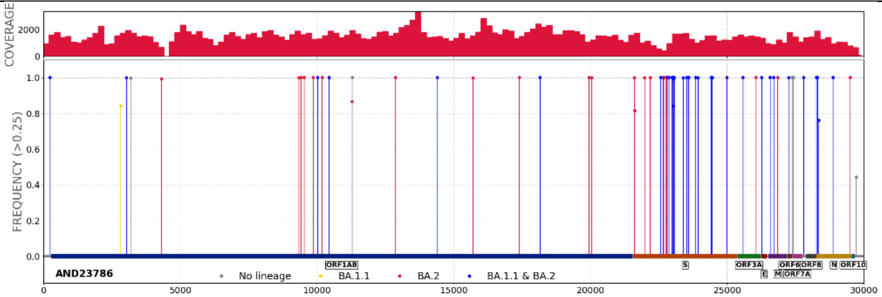

Breakpoint close to XQ (4322-5385) or XR (4322-4891) recombinant</td></tr>
</table>

AND23787	recombinant / XQ (BA.1.1-BA.2)	BA.2	4184-4321 (nsp3)	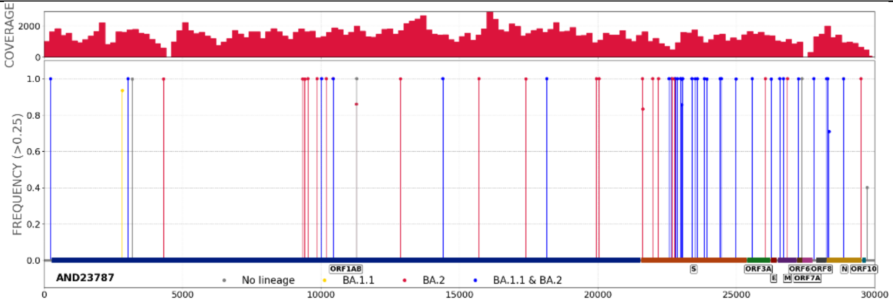<br>Breakpoint close to XQ (4322-5385) or XR (4322-4891) recombinant
AND23791	recombinant / XQ (BA.1.1-BA.2)	BA.2	4184-4321 (nsp3)	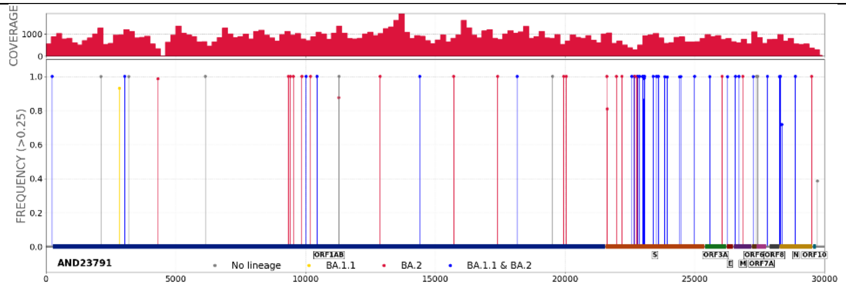<br>Breakpoint close to XQ (4322-5385) or XR (4322-4891) recombinant

AND24298	recombinant / XQ (BA.1.1-BA.2)	BA.2	4184-4321 (nsp3)	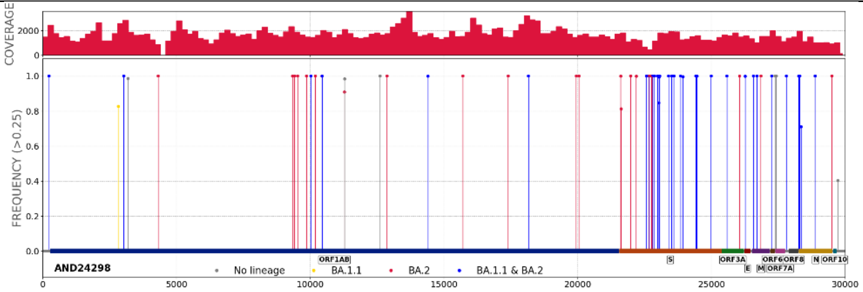<br>Breakpoint close to XQ (4322-5385) or XR (4322-4891) recombinant
AND24301	recombinant / XQ (BA.1.1-BA.2)	BA.2	4184-4321 (nsp3)	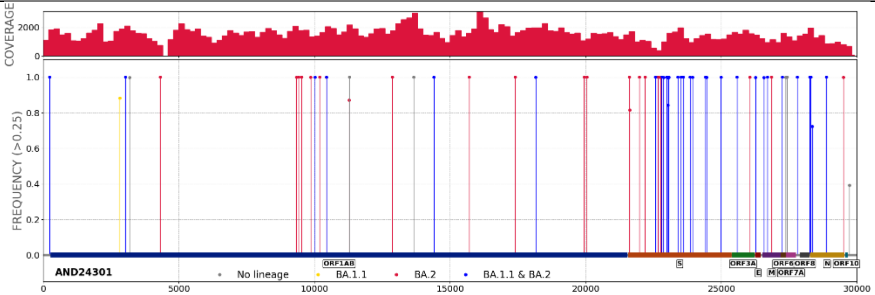<br>Breakpoint close to XQ (4322-5385) or XR (4322-4891) recombinant

AND24302	recombinant / XQ (BA.1.1-BA.2)	BA.2	4184-4321 (nsp3)	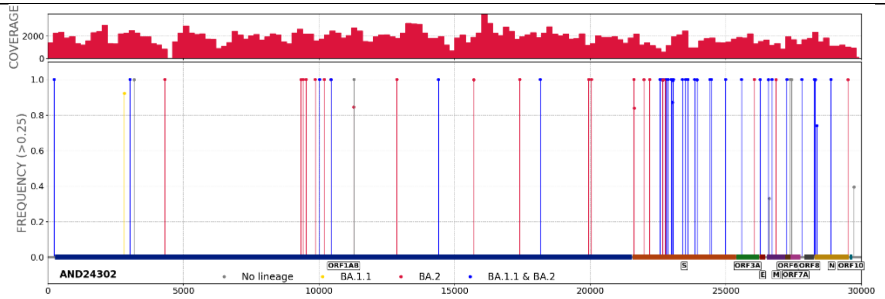 Breakpoint close to XQ (4322-5385) or XR (4322-4891) recombinant
AND24350	recombinant / XN (BA.1-BA.2)	BA.2	3037-4184 (nsp3)	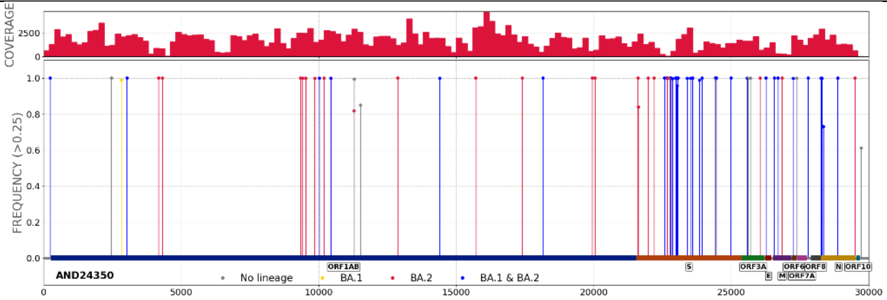 Breakpoint close to XN (2834-4183)

AND24357	recombinant / XT (BA.1-BA.2)	BA.2	25584-26060 (S or ORF3a)	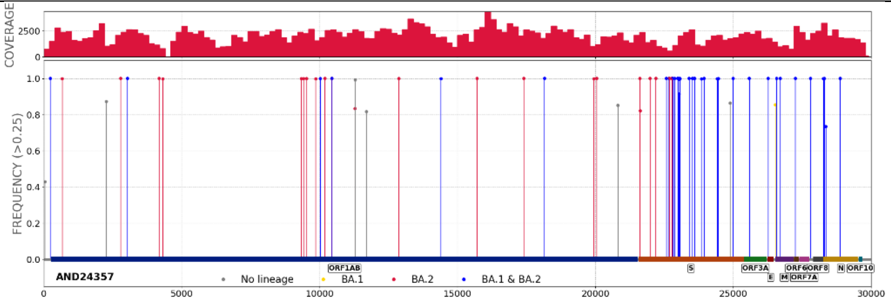 Breakpoint close to XT (26062-26528) and XAE (24506-26048)
AND24658	recombinant / XN (BA.1-BA.2)	BA.2	3037-4184 (nsp3)	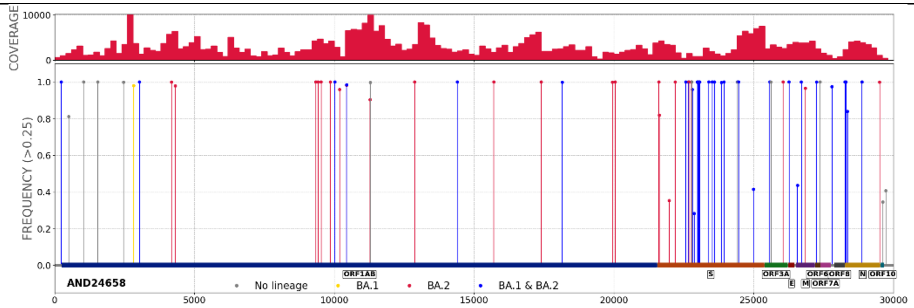 Breakpoint close to XN (2834-4183)

AND24834	recombinant / XQ (BA.1.1-BA.2)	BA.2	4184-4321 (nsp3)	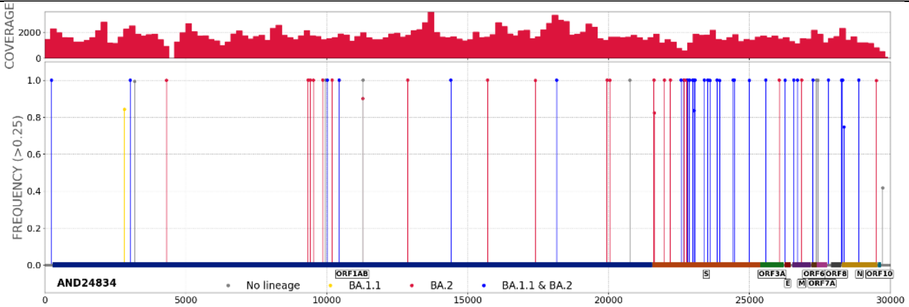 Breakpoint close to XQ (4322-5385) or XR (4322-4891) recombinant
AND24843	recombinant / XN (BA.1-BA.2)	BA.2	3037-4184 (nsp3)	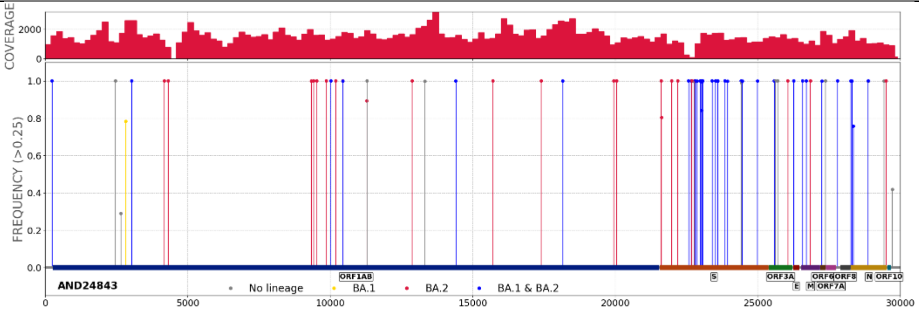 Breakpoint close to XN (2834-4183)

AND24864	recombinant / XQ (BA.1.1-BA.2)	BA.2	4184-4321 (nsp3)	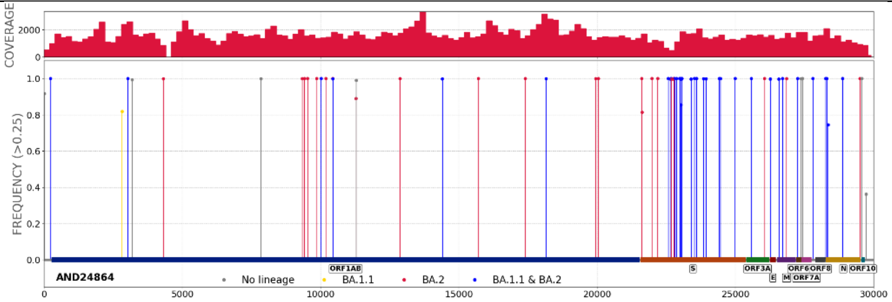<br>Breakpoint close to XQ (4322-5385) or XR (4322-4891) recombinant
AND24884	recombinant / XQ (BA.1.1-BA.2)	BA.2	4184-4321 (nsp3)	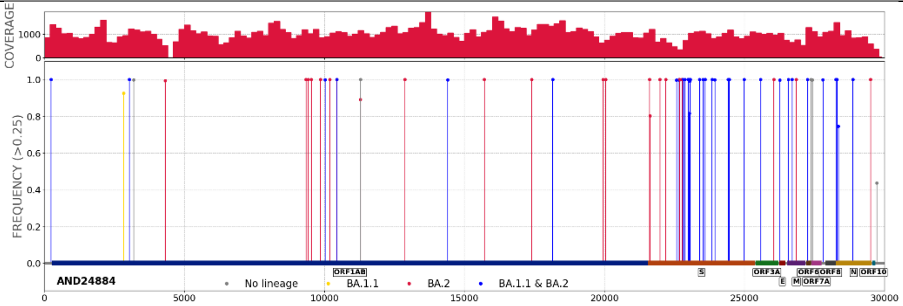<br>Breakpoint close to XQ (4322-5385) or XR (4322-4891) recombinant

AND25258	recombinant / XQ (BA.1.1-BA.2)	BA.2	4184-4321 (nsp3)	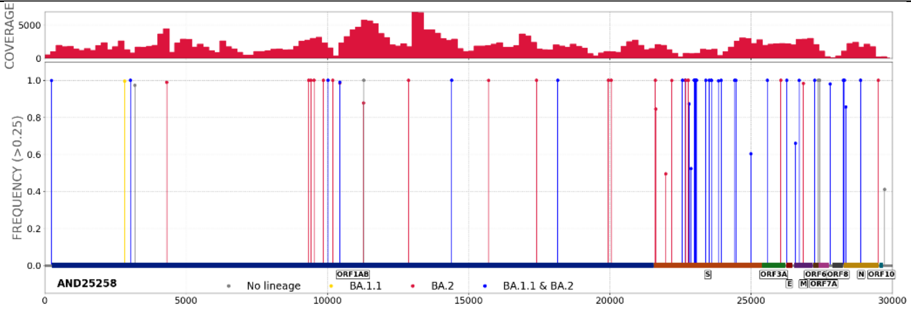<br>Breakpoint close to XQ (4322-5385) or XR (4322-4891) recombinant

Table S4. Sequences of the family in which the emergence of a recombinant was observed

Sample	Event (C=Coinfection, R=Recombinant, D=Delta VOC)	Collection date	Localization (Hospital and Province)	ENA Sample ID
AND18640	D	2021-12-28	Hospital Universitario Reina Sofía (Córdoba)	SAMEA110403704
AND19730*	D	2022-02-02	Hospital Universitario Reina Sofía (Córdoba)	SAMEA110403705
AND20071*	C	2022-02-09	Hospital Universitario Reina Sofía (Córdoba)	SAMEA110403706
AND21237*	R	2022-03-02	Hospital Universitario Reina Sofía (Córdoba)	SAMEA110395597
AND23187*	R	2022-02-18	Hospital Universitario Reina Sofía (Córdoba)	SAMEA110395633
AND23055*	R	2022-03-02	Hospital Universitario Reina Sofía (Córdoba)	SAMEA110395673
AND23277*	D	2021-12-30	Hospital Universitario Reina Sofía (Córdoba)	SAMEA110403707
AND24267	D	2022-02-04	Hospital Universitario Reina Sofía (Córdoba)	SAMEA110403708

Table S5. Detailed Lolliplots of the sequences of the family in which the emergence of a recombinant was observed

<table>
<tr><th>Sample</th><th>Event

(C=Coinfection,

R=Recombinant,
D=Delta VOC)</th><th>Lollipop plot</th></tr>
<tr><td>AND18640</td><td>D</td><td>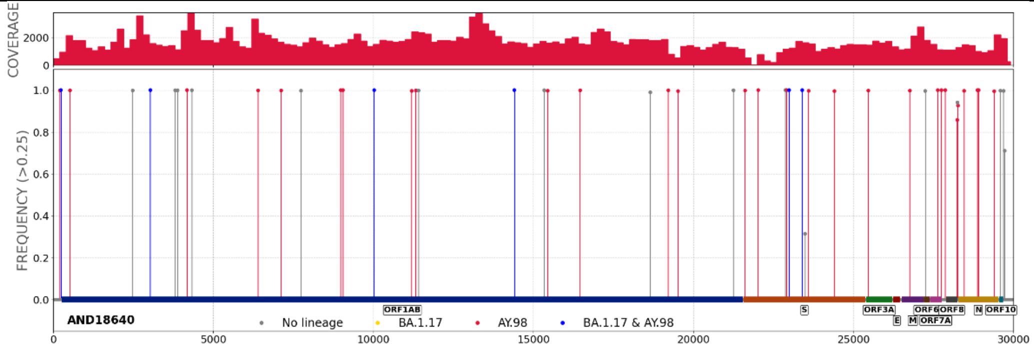
</td></tr>
<tr><td>AND19730*</td><td>D</td><td>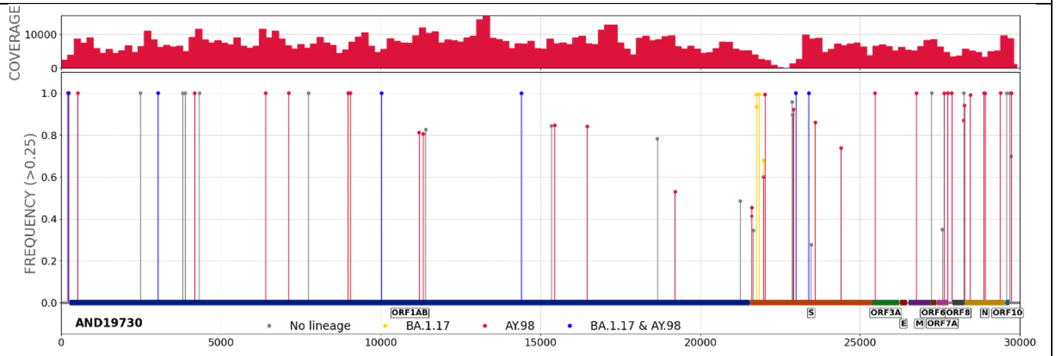
</td></tr>
</table>

AND20071*	C	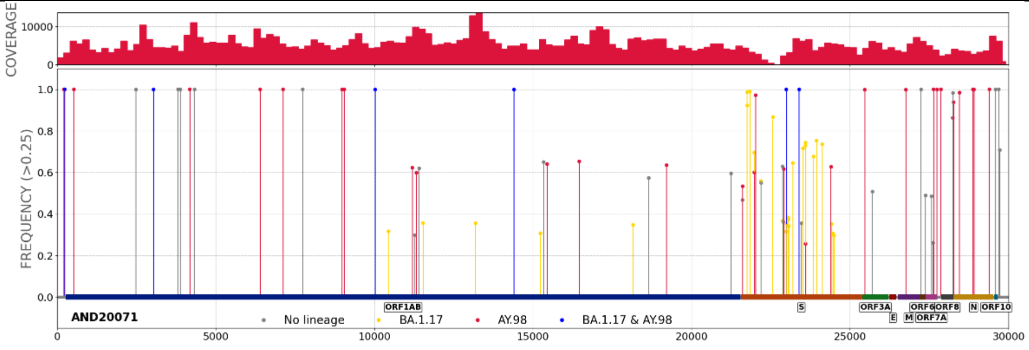
AND21237*	R	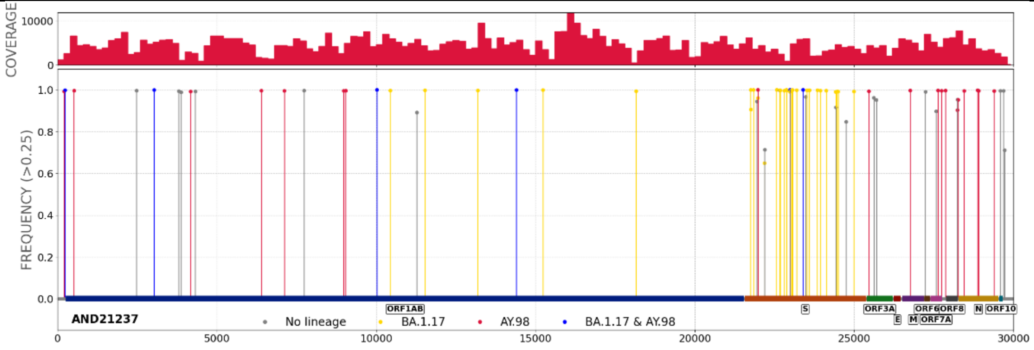
AND23187*	R	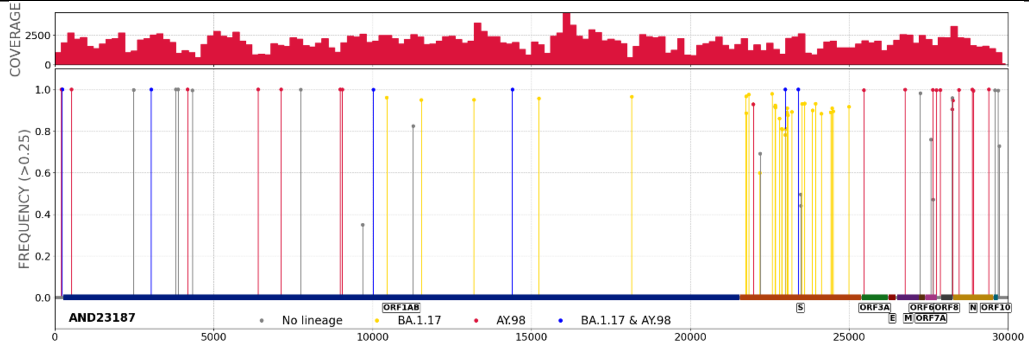

AND23055*	R	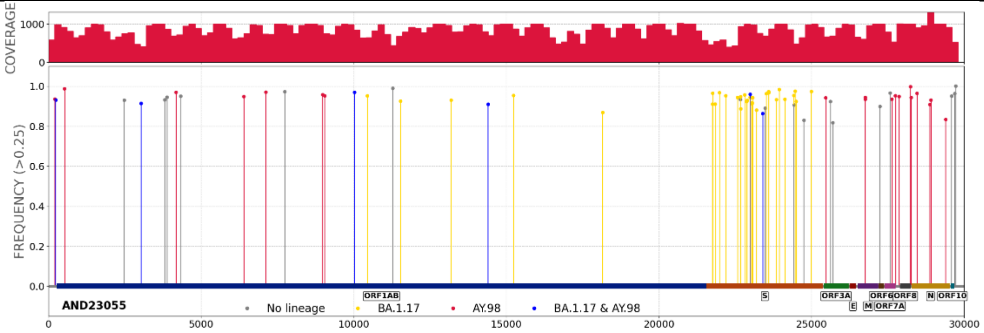
AND23277*	D	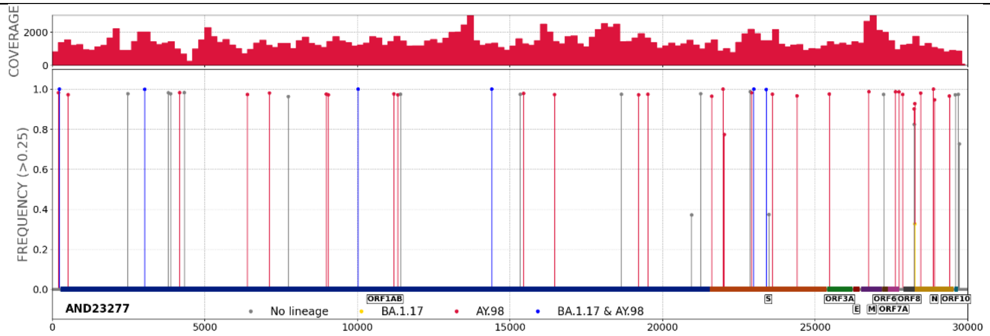
AND24267	D	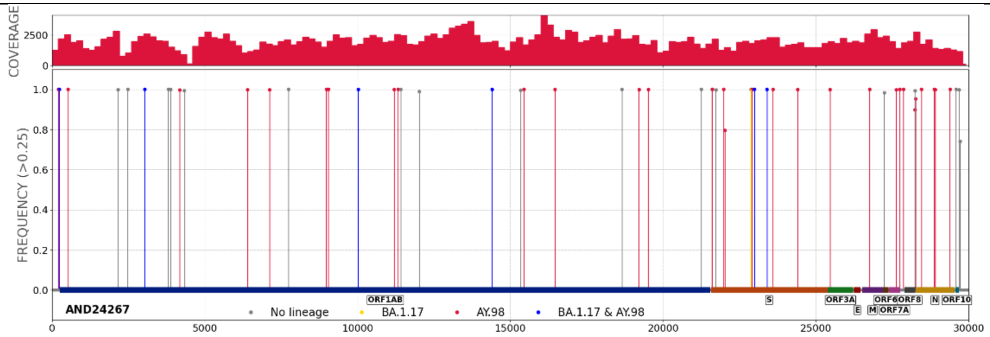

The Andalusian COVID-19 Sequencing Initiative

Francisco J. Morón, **Rosana March-Diaz, Salud Borrego, Irene Marcos**: Institute of Biomedicine of Seville (IBIS), Hospital Virgen del Rocio, 41013 Sevilla, Spain

Mónica Perez-Alegre, Eloísa Andújar: Centro Andaluz de Biología Molecular y Medicina Regenerativa CABIMER, Universidad de Sevilla-CSIC-Universidad Pablo de Olavide, Sevilla, Spain

Matilde Palanca Giménez: Hospital Poniente de Almería, El Ejido, Almería, Spain

Teresa Cabezas, Manuel Rodríguez Maresca: Hospital Torrecárdenas, Almería, Spain

Manuel Causse del Río, Luis Martínez-Martínez: Hospital Universitario Reina Sofía, Córdoba, Spain

Francisco Franco Álvarez De Luna: Hospital Juan Ramón Jiménez, Huelva, Spain

Carolina Roldán Fontana: Complejo Hospitalario de Jaén, Jaén, Spain

María Dolores López Prieto: Hospital de Jerez, Cádiz, Spain

Maria Luisa Hortas, Fernando Fernández Sánchez: Hospital Costa del Sol, Málaga, Spain

Begoña Palop Borrás: Hospital Regional, Málaga, Spain

Isabel Viciana: Hospital Virgen de la Victoria, Málaga, Spain

Alvaro Pascual: Hospital Virgen de la Macarena, Sevilla; Institute of Biomedicine of Seville (IBIS), Hospital Virgen del Rocio, 41013 Sevilla, Spain

Ángel Rodríguez Villodres: Hospital Universitario Virgen del Rocío, Sevilla, Spain

Samuel Bernal Martinez, Estrella Martin Mazuelos: Unidad Clínica de Enfermedades Infecciosas y Microbiología (UCEIM), H.U. Virgen de Valme, Sevilla, Spain

Inés Ruiz Molina: Hospital Punta Europa, 11207, Cádiz, Spain

Jesus Rodriguez-Baño: Institute of Biomedicine of Seville (IBIS), Hospital Virgen del Rocio, 41013 Sevilla, Spain; Microbiología y Medicina Preventiva, Hospital Universitario Virgen Macarena, 41009 Sevilla, Spain; Departamento de Medicina, Universidad de Sevilla, 41004 Sevilla, Spain; Centro de Investigación Biomédica en Red en Enfermedades Infecciosas (CIBERINFEC), ISCIII, Madrid, Spain

Nicola Lorusso: Dirección General de Salud Pública. Consejería de Salud y Familias. Junta de Andalucía, 41020 Sevilla, Spain

Javier Garcia-León: Departamento de Metafísica y Corrientes Actuales de la Filosofía, Ética y Filosofía Política, Universidad de Sevilla, 41004 Sevilla, Spain

Jose M. Navarro-Marí: Servicio de Microbiología, Hospital Virgen de las Nieves, 18014 Granada, Spain. Instituto de Investigación Biosanitaria, ibs.GRANADA, 18012 Granada, Spain

L. Javier Martínez-González: GENYO. Centre for Genomics and Oncological Research, Pfizer/University of Granada/Andalusian Regional Government, PTS Granada, 18016 Granada, Spain

Dolores Muñoyerro-Muñiz, Román Villegas: Subdirección Técnica Asesora de Gestión de la Información, Servicio Andaluz de Salud, 41001 Sevilla, Spain
